# A Hybrid Lifestyle Program and Metabolic Health in Adults With Obesity: The PRAMEV Randomized Clinical Trial

**DOI:** 10.1001/jamanetworkopen.2026.26884

**Published:** 2026-08-11

**Authors:** Graziele Souza de Menezes Amorim Coelho, Ariana Ester Fernandes, Renata Bressan Pepe, Patrícia Aparecida Cruz, Andrea Samara Audi, Cintia Cercato, Marcio C. Mancini, Maria Edna de Melo

**Affiliations:** 1 Grupo de Obesidade e Sindrome Metabolica, Hospital das Clinicas HCFMUSP, Faculdade de Medicina, Universidade de Sao Paulo, Sao Paulo, SP, Brazil; 2 Unidade de Medicina Interdisciplinar em Cardiologia, Instituto do Coração, Hospital das Clínicas HCFMUSP, Faculdade de Medicina, Universidade de São Paulo, São Paulo, Brazil; 3 Laboratório de Carboidratos e Radioimunoensaios (LIM 18), Faculdade de Medicina FMUSP, Universidade de São Paulo, São Paulo, SP, Brazil; 4 Laboratório de Lipides (LIM 10), Hospital das Clínicas HCFMUSP, Faculdade de Medicina, Universidade de São Paulo, São Paulo, SP, Brazil

## Abstract

**Importance:**

Obesity disproportionately affects low- and middle-income countries, where access to structured, scalable treatment remains limited.

**Objective:**

To evaluate whether a hybrid-format, intensive lifestyle modification program combined with usual medical care promotes a minimum weight loss of 5% in adults with obesity compared with usual medical care alone.

**Design, Setting, and Participants:**

This open-label, parallel-group, superiority randomized clinical trial recruited adults with a body mass index of 30 or greater from a tertiary public hospital in São Paulo, Brazil, between January 1 and March 31, 2022, randomized 1:1 to PRAMEV (Advanced Program for Intensive Lifestyle Modification) or control. Data were analyzed from May to September 2025 using an intention-to-treat approach.

**Interventions:**

PRAMEV was a 20-week, hybrid lifestyle intervention that included group-based behavioral counseling, nutritional education, physical activity promotion, and continuous instructor support. The control group received usual medical care.

**Main Outcomes and Measures:**

The primary outcome was the proportion of participants achieving a 5% or greater reduction in body weight assessed by bioelectrical impedance analysis at 20 weeks. Secondary outcomes included changes in weight, body mass index, waist circumference, metabolic parameters (lipid profile, glucose, and liver enzyme levels), dietary intake, and psychosocial variables.

**Results:**

A total of 200 participants (mean [SD] age, 47.9 [14.0] years; 172 [86.0%] women) were randomized to PRAMEV (n = 97) or control (n = 103). At 20 weeks, 143 participants (71.4%) completed follow-up, with higher dropout in the PRAMEV arm vs the control arm (39.2% [38 of 97] vs 21.4% [22 of 103]; *P* = .008). In the intention-to-treat analysis, groups did not differ in achieving 5% or greater weight loss (20.6% [20 of 97] vs 15.5% [16 of 103]; odds ratio, 1.34; 95% CI, 0.61-2.92; *P* = .46). Per-protocol analyses showed greater weight loss and improved anthropometric outcomes in the PRAMEV arm. The intervention improved the lipid profile, with reductions in liver enzyme and triglyceride levels and favorable changes in cholesterol fractions. No serious adverse events were reported.

**Conclusions and Relevance:**

In this randomized clinical trial of 200 adults with obesity receiving care in Brazil’s public health care system, a hybrid-format lifestyle intervention did not improve the likelihood of achieving 5% or greater weight loss in the intention-to-treat analysis. However, secondary and per-protocol analyses suggested potential cardiometabolic benefits among adherent participants, warranting further investigation.

**Trial Registration:**

ReBEC Identifier: U1111-1273-8313

## Key Points

**Question:**

Can a hybrid-format, intensive lifestyle intervention integrated into a public health care system promote a minimum weight loss of 5% in adults with obesity compared with usual medical care alone?

**Findings:**

In this randomized clinical trial of 200 participants, the 20-week hybrid lifestyle program did not significantly increase the proportion achieving 5% or greater weight loss compared with usual medical care in the intention-to-treat analysis. However, improvements in metabolic markers and per-protocol analyses suggested potential benefits among adherent participants.

**Meaning:**

This study’s results suggest that a culturally adapted, scalable lifestyle intervention can be integrated into public health care, and adherence may confer cardiometabolic benefits beyond pharmacologic treatment alone.

## Teaser

This randomized clinical trial of individuals with obesity assesses whether a hybrid, intensive lifestyle intervention combined with usual medical care promotes weight loss.

## Introduction

Obesity is a major global health challenge, affecting more than 890 million adults worldwide and contributing substantially to type 2 diabetes, cardiovascular disease, and cancer.^1,2,3^ Obesity also imposes a substantial economic burden on health care systems, driven by hospitalizations, medication use for chronic diseases, surgical procedures, and management of comorbidities.^4,5^

Despite available pharmacologic and surgical options, sustained weight loss remains difficult to achieve at the population level.^6,7^ Obesity is a complex, multifactorial condition, and lifestyle modification remains the cornerstone of treatment across all therapeutic pathways.^8,9,10,11^ Intensive lifestyle interventions, such as those modeled after the Diabetes Prevention Program, have proven effective in controlled trials.^12,13,14,15,16^ However, implementation in community settings, especially in low- and middle-income countries, is limited by resource constraints, workforce shortages, and logistical barriers.^17^

In Brazil, nearly 25% of adults live with obesity, and prevalence continues to increase across socioeconomic groups.^18^ Within the public health care system Sistema Único de Saúde, one of the largest in the world, access to comprehensive obesity care remains limited.^19^ Less than 3% of primary care visits address obesity,^20^ and existing services often rely on nonstandardized or low-efficacy strategies. Health professionals also receive limited training in evidence-based obesity management.

To address these limitations, scalable and pragmatic models of care are needed. Hybrid, intensive lifestyle interventions that combine in-person and remote components have emerged as a promising strategy, offering flexibility, cost efficiency, and broader reach, particularly in the post–COVID-19 era.^21^ The PRAMEV (Advanced Program for Intensive Lifestyle Modification) program was designed with a 5% weight loss target for participants. Our hypothesis was that a higher proportion of participants receiving the PRAMEV intervention in conjunction with usual care would achieve this target compared with those receiving usual care alone.

## Methods

### Study Design and Participants

This open-label, parallel-group, superiority randomized clinical trial (1:1 ratio) was conducted at a tertiary public hospital in São Paulo, Brazil. This study was approved by the University of São Paulo Ethics Committee, registered in the Brazilian Clinical Trials Registry, and conducted in accordance with the Declaration of Helsinki^22^ and Consolidated Standards of Reporting Trials (CONSORT) guidelines.^23^ All participants provided written informed consent.

Participants were recruited from the Obesity Outpatient Clinic at the Hospital das Clínicas, University of São Paulo Medical School, from January 1 to March 31, 2022. The full trial protocol and statistical analysis plan are available in Supplement 1. The trial was completed as planned with no early termination. Patients and the public were not involved in the design, conduct, or reporting of the study. Protocol versions 1.1 and 1.2 included pretrial adjustments to the program name, randomization, and adherence monitoring, with no relevant changes after initiation. Race was self-reported using the 5 color or race categories of the Instituto Brasileiro de Geografia e Estatística (Brazilian Institute of Geography and Statistics): Asian, Black, Indigenous, White, and multiracial (individuals of mixed Black, Indigenous, and/or White ancestry).^24^ Race data were collected as part of standard demographic characterization of the study sample, allowing assessment of sample diversity and representativeness, and evaluation of equitable access to the intervention across racial groups within Brazil’s public health care system.

Eligible participants were adults (aged ≥18 years) with body mass index (BMI) of 30 or greater (calculated as weight in kilograms divided by height in meters squared) who were literate and capable of accessing telemedicine. Exclusion criteria included previous bariatric surgery, uncontrolled endocrine or psychiatric disorders, genetic obesity syndromes, cognitive impairment, and, for women, pregnancy or lactation.

The sample size calculation defined absolute weight loss as the primary outcome. Based on a prior lifestyle intervention study,^25^ we assumed a mean (SD) weight loss of 5 (5) kg in the PRAMEV group and 2 (2) kg in the control group (Cohen *d* = 0.79) to be clinically relevant. A total of 27 participants per group provided 80% power at a 5% significance level (2-sided; G*Power, version 3.1.9.2, University of Dusseldorf).

The trial was designed to be pragmatic, aiming to evaluate the intervention’s effects under usual clinical conditions rather than idealized circumstances: participants were recruited directly from a routine endocrinology clinic, minimal exclusion criteria were applied, and patients with prior weight-loss attempts and those already receiving treatment, whether pharmacologic or lifestyle counseling, were eligible, reflecting the heterogeneity of patients typically seen in routine clinical practice. Given the expected attrition in behavioral trials, all 457 clinic patients during the recruitment period were invited. Of these, 317 were screened, and 200 eligible participants were randomized to PRAMEV or control. Participant flow is shown in the Figure. No interim analyses or stopping rules were planned. Randomization was performed using the RAND function in Microsoft Excel (Microsoft Corp), stratified by BMI. The sequence was generated before recruitment and applied sequentially after eligibility confirmation. Allocation was individual and disclosed after informed consent. The slight group imbalance (97 in the PRAMEV group and 103 in the control group) reflects random variation.

**Figure.**
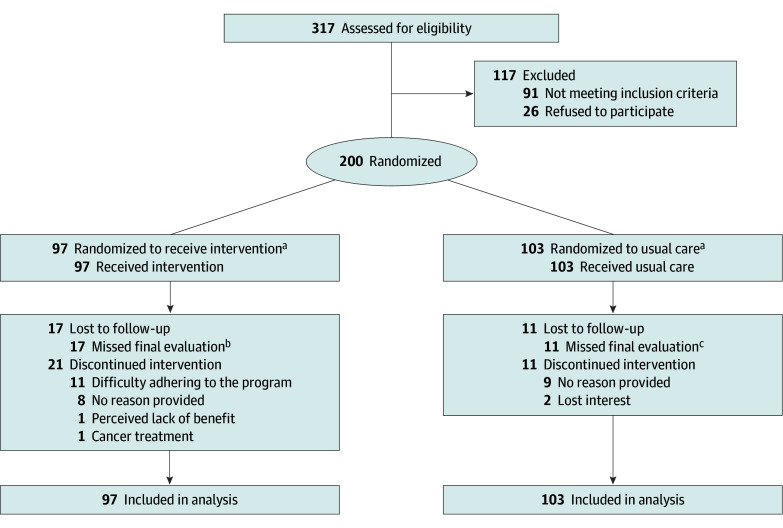
Study Flow Diagram ^a^Participants in both groups received usual medical care. The intervention group additionally received the PRAMEV (Advanced Program for Intensive Lifestyle Modification) intervention. ^b^Reasons for missing the final evaluation among 17 participants were as follows: no reason provided (n = 9), logistical constraints (n = 3), lack of transportation (n = 3), hospitalization (n = 1), and inability to obtain leave from work (n = 1). ^c^Reasons for missing the final evaluation among 11 participants were as follows: no reason provided (n = 5), lack of transportation (n = 2), scheduling conflict (n = 1), inability to obtain leave from work (n = 1), illness (n = 1), and hospitalization (n = 1).

Blinding of outcome assessors was not feasible given the open-label behavioral nature of the intervention. To reduce detection bias, objective measurements and standardized assessment protocols were used.

Both groups underwent in-person assessments at baseline and week 20 and continued usual endocrinology care quarterly. PRAMEV additionally received weekly online guidance. Further details are provided in the protocol (Supplement 1).

### Assessments

Weight, height, and waist circumference were measured using standardized protocols at baseline and week 20. Details are described in Supplement 1. Body composition and basal metabolic rate were assessed using bioelectrical impedance analysis (InBody 720, Biospace Co Ltd). Additional parameters included blood pressure, heart rate, and biochemical tests (total cholesterol, low-density lipoprotein cholesterol [LDL-C], high-density lipoprotein cholesterol [HDL-C], triglycerides, alanine aminotransferase [ALT], aspartate aminotransferase [AST], fasting glucose, glycated hemoglobin, and insulin levels). Physical activity was self-reported by type, duration, and intensity, and energy expenditure was estimated using metabolic equivalent of task (MET) values (calculated as MET × weight [in kilograms] × duration [in hours]).^26^

Dietary intake was assessed using 2-day food records at baseline and week 20. Baseline and final records were compared and analyzed with Dietbox software, version 1.9.26 (Dietbox) to estimate energy and macronutrient intake. Foods were classified by NOVA (unprocessed/minimally processed, processed culinary ingredients, processed, or ultraprocessed).^27^

The Binge Eating Scale was used to assess binge eating^28^ and the Depression, Anxiety, and Stress Scale to evaluate related symptoms.^29^ Both were validated for Portuguese-speaking populations.

Body image perception was assessed by asking participants to report desired weight at baseline and after the 20-week intervention. The difference between current and desired weight was calculated at both time points, and a reduction was interpreted as a shift toward a more realistic body image.

### Intervention

Participants assigned to PRAMEV followed a culturally adapted version of the Diabetes Prevention Program^30^ for implementation within Brazil’s public health system. The intervention was delivered in a hybrid format, combining weekly online and in-person group sessions to improve accessibility and reduce transportation barriers for patients receiving care in a large public hospital. The program included standardized guidance on diet, physical activity, and self-monitoring. Nutritional advice emphasized calorie reduction without prescribed diets, using behavioral strategies aligned with local dietary habits and the Dietary Guidelines for the Brazilian Population.^31^ Physical activity targets were 150 or more minutes per week of moderate-to-vigorous exercise.

Communication with participants was conducted through the WhatsApp application (Meta Platforms), using simplified behavioral strategies adapted for individuals with varying educational levels. The intervention was integrated into routine care and delivered alongside usual endocrinology follow-up without additional infrastructure.

Adherence was monitored weekly using a 3-point self-assessment scale, with feedback from trained lifestyle instructors (physicians or nutritionists) who received standardized training before the study. A messaging-based support system was maintained throughout the intervention. In the control group, adherence was defined as attendance at the week 20 follow-up assessment. Further details are available in Supplement 1.

### Outcome Measures

The primary outcome was the proportion of participants achieving a 5% or greater reduction in body weight, assessed by bioelectrical impedance analysis, from baseline to week 20. Secondary outcomes included changes in body weight (kilograms and percentage), BMI, waist circumference, metabolic parameters (lipid profile, glucose, and liver enzyme levels), body composition, basal metabolic rate, physical activity energy expenditure (MET in hours per day), dietary intake (kilocalories per day, macronutrient composition, and food processing level according to the NOVA classification), and psychosocial variables (body image perception, binge eating symptoms, and emotional distress).

Exploratory outcomes included antiobesity medication use, session attendance, and dropout rates, with reasons for discontinuation and variation by age, sex, BMI, body weight, waist circumference, and program attendance. Age was also analyzed by generational cohort to assess differences in dropout patterns, defined as Baby Boomers (1946-1964), Generation X (1965-1980), Generation Y or Millennials (1981-1996), and Generation Z (1997-2012). No adverse events were reported, and monitoring details are provided in Supplement 1.

### Statistical Analysis

Data normality was assessed using the Shapiro-Wilk test. Baseline characteristics were summarized as means (SDs), medians (IQRs), or numbers (percentages). The primary analysis followed the intention-to-treat (ITT) principle, including all randomized participants. Missing data at week 20 were handled using multiple imputation by chained equations (n = 50) under the missing at random assumption, with estimates pooled using Rubin rules.

The primary outcome was analyzed using logistic regression adjusted for baseline BMI, age, and sex, with results expressed as odds ratios (ORs) and 95% CIs. Continuous anthropometric outcomes (weight in kilograms, percentage weight, BMI, and waist circumference) were analyzed as change from baseline using linear regression models adjusted for baseline values, age, and sex. Other secondary outcomes were compared between groups using a 2-sided Student *t* test or the Mann-Whitney *U* test, as appropriate.

Sensitivity analyses under missing not at random assumptions were performed for the primary and anthropometric outcomes using a tipping point approach, with progressive penalties applied to participants with missing data in the intervention group. A secondary per-protocol analysis included participants who completed the 20-week intervention, regardless of missing data handling.

Associations between session adherence (as percentage attended) and changes were examined using Spearman correlation, with bootstrapped 95% CIs (1000 resamples). Exploratory within-group comparisons were conducted using paired *t* tests or Wilcoxon signed-rank tests. Subgroup analyses by generational cohort evaluated dropout rate differences using χ^2^ tests.

All analyses were performed using Python, version 3.11 (Python Software Foundation), with a 2-sided significance threshold of *P* < .05. Data were analyzed from May to September 2025.

## Results

### Baseline Characteristics and Demographics

A total of 200 adults with obesity (mean [SD] age, 47.9 [14.0] years; 172 [86.0%] women; 2 [1.0%] Asian, 25 [12.5%] Black, 161 [80.5%] White, and 12 [6.0%] multiracial) were randomized to the PRAMEV (n = 97) or control (n = 103) group. A total of 184 (92.5%) reported low to middle incomes (<5 Brazilian minimum wages) and 41 (20.7%) had an undergraduate degree (Table 1). At baseline, both groups showed similar median (IQR) values for weight (95.4 [83.3-106.5] vs 95.9 [88.4-108.4] kg), BMI (37.0 [33.8-41.1] vs 37.2 [33.7-41.1]), waist circumference (113.8 [105.4-121.3] vs 113.1 [105.3-122.0] cm), and body fat percentage (50.0% [45.6%-52.5%] vs 48.7% [45.6%-51.3%]) (Table 2). At baseline, participants showed mild depression and stress and moderate anxiety across both groups. Median (IQR) Binge Eating Scale scores were 12.0 (7.0-20.0) in the PRAMEV group and 12.0 (5.5-18.0) in the control group, consistent with non–binge-eating behavior (scores ≤17).^28^ Baseline laboratory and dietary characteristics are given in Table 2.

**Table 1.** Baseline Demographic Characteristics of Study Participants

Characteristic^a^^,^^b^	No. (%) of participants^c^	
	PRAMEV group (n = 97)	Control group (n = 103)
Age, mean (SD), y	49.4 (14.5)	46.5 (13.4)
Sex		
Female	79 (81.4)	93 (90.3)
Male	18 (18.6)	10 (9.7)
Race^d^		
Asian	2 (2.1)	0 (0.0)
Black	16 (16.5)	9 (8.7)
White	74 (76.3)	87 (84.5)
Multiracial	5 (5.2)	7 (6.8)
Educational attainment, No./total No. (%)^e^		
Elementary school or less	31/96 (32.0)	38/102 (36.9)
High school graduate	46/96 (47.4)	42/102 (40.8)
Undergraduate degree or higher	19/96 (19.6)	22/102 (21.4)
Marital status		
Married	43 (44.3)	51 (49.5)
Single, divorced, or widowed	54 (45.4)	52 (41.8)
Monthly household income, in minimum wages, No./total No. (%)^f^^,^^g^^,^^h^		
No income	1/97 (1.0)	4/102 (3.9)
≤1 Minimum wage	15/97 (15.5)	13/102 (12.8)
>1 to <2 Minimum wages	32/97 (33.0)	42/102 (41.2)
≥2 to <5 Minimum wages	40/97 (41.2)	37/102 (36.3)
≥5 to <10 Minimum wages	8/97 (8.3)	4/102 (3.9)
≥10 to <30 Minimum wages	1/97 (1.0)	2/102 (2.0)

Abbreviation: PRAMEV, Advanced Program for Intensive Lifestyle Modification.

^a^ Data are from electronic health records at the time of recruitment.

^b^ Self-reported data were used to supplement missing electronic health record values.

^c^ Unless otherwise indicated.

^d^ No participants self-identified as Indigenous.

^e^ The number of participants for the educational attainment category represents nonmissing values, which serve as the denominator for subsequent percentages. Missing data are due to participant refusal.

^f^ Monthly household income is expressed in multiples of the Brazilian minimum wage in effect in 2022.

^g^ Based on self-report only.

^h^ The number of participants for the income category represents nonmissing values, which serve as the denominator for subsequent percentages. Missing data occurred only in the control group due to participant refusal.

**Table 2.** Baseline Clinical, Behavioral, and Dietary Characteristics of Study Participants

Characteristic	PRAMEV group (n = 97)^a^	Control group (n = 103)^a^
Weight, kg	95.4 (83.3-106.5)	95.9 (88.4-108.4)
Maximum weight, kg	106.0 (94.0-120.0)	107.0 (98.0-122.5)
Prior weight loss, %	7.6 (3.5-14.3)	9.3 (2.7-14.5)
Desired weight, kg	70.0 (60.0-75.0)	70.0 (65.0-75.0)
BMI	37.0 (33.8-41.1)	37.2 (33.7-41.1)
WC, cm	113.8 (105.4-121.3)	113.1 (105.3-122.0)
SBP, mm Hg	126.5 (115.3-137.8)	120.0 (112.0-140.0)
DBP, mm Hg	80.0 (76.3-90.0)	80.0 (70.0-90.0)
HR, beats/min	79.2 (16.9)	78.9 (11.4)
TEE, kcal/d	1423.0 (1296.0-1564.0)	1457.0 (1365.0-1568.0)
SMM, kg	26.4 (23.2-30.8)	27.6 (25.3-31.0)
BF, %	50.0 (45.6-52.5)	48.7 (45.6-51.3)
Physical activity energy expenditure, kcal/d	0.0 (0.0-265.0)	0.0 (0.0-223.7)
AST, U/L	20.0 (14.0-29.0)	18.0 (14.0-25.0)
ALT, U/L	19.0 (16.0-24.8)	18.0 (16.0-23.0)
Glucose, mg/dL	94.0 (87.0-105.0)	96.0 (88.0-106.0)
HbA_1c_, %	5.6 (5.2-6.2)	5.6 (5.3-6.2)
LDL-C, mg/dL	107.5 (91.5-130.3)	104.5 (83.8-126.0)
HDL-C, mg/dL	49.0 (40.0-56.0)	48.0 (40.0-56.0)
Serum total cholesterol, mg/dL	182.0 (155.0-209.0)	176.0 (154.0-197.0)
Triglycerides, mg/dL	113.0 (82.0-144.0)	119.0 (87.0-150.0)
Stress score	16.0 (6.0-26.0)	18.0 (10.0-28.0)
Depression score	10.0 (4.0-24.0)	12.0 (6.0-25.0)
Anxiety score	12.0 (4.0-18.0)	12.0 (4.0-22.0)
BES score	12.0 (7.0-20.0)	12.0 (5.5-18.0)
Use of antiobesity pharmacotherapy, No. (%)	73 (75.3)	77 (74.8)
Total energy intake, kcal	1345.2 (1143.5-1650.2)	1345.3 (1189.2-1734.5)
Total protein, % TEI	18.4 (5.4)	17.8 (14.6-20.6)
Total carbohydrate, % TEI	49.2 (9.5)	49.4 (43.3-55.4)
Total lipid, % TEI	31.1 (6.2)	31.6 (26.7-39.6)
Saturated fat, % TEI	10.6 (2.4)	11.9 (9.2-14.1)
Monounsaturated fat, % TEI	10.0 (3.2)	9.1 (7.9-12.9)
Polyunsaturated fat, % TEI	6.2 (4.7-7.9)	6.5 (2.1)
Dietary cholesterol, mg/dL	224.7 (154.3-320.0)	193.1 (153.5-351.1)
Total fiber, g	13.2 (9.3-17.9)	15.3 (9.9-20.7)
G1, % TEI	58.7 (18.1)	55.1 (19.3)
G2, % TEI	2.3 (0.6-6.2)	2.0 (0.1-6.3)
G3, % TEI	12.1 (4.3-20.5)	15.1 (8.5-30.6)
G4, % TEI	22.4 (13.3)	23.2 (10.1-32.9)

Abbreviations: ALT, alanine aminotransferase; AST, aspartate aminotransferase; BF, body fat; BES, Binge Eating Scale; BMI, body mass index (calculated as weight in kilograms divided by height in meters squared); DBP, diastolic blood pressure; G1, group 1 (unprocessed or minimally processed foods); G2, group 2 (processed culinary ingredients); G3, (processed foods); G4, (ultraprocessed foods and beverages); HbA_1c_, glycated hemoglobin; HDL-C, high-density lipoprotein cholesterol; HR, heart rate; LDL-C, low-density lipoprotein cholesterol; PRAMEV, Advanced Program for Intensive Lifestyle Modification; SBP, systolic blood pressure; SMM, skeletal muscle mass; TEE, total energy expenditure; TEI, total energy intake; WC, waist circumference.

SI conversion factors: To convert glucose to millimoles per liter, multiply by 0.0555; cholesterol to millimoles per liter, multiply by 0.0259; triglycerides to millimoles per liter, multiply by 0.0113; AST and ALT to microkatals per liter, multiply by 0.0167.

^a^ Data are presented as median (IQR) or mean (SD) unless otherwise indicated.

### Primary Outcome

In the ITT analysis, the PRAMEV and control groups did not differ in achieving 5% or greater weight loss (20 of 97 [20.6%] vs 16 of 103 [15.5%]; OR, 1.34; 95% CI, 0.61-2.92; *P* = .46), with consistent results in missing not at random sensitivity analyses (eTable 1 in Supplement 2). In the per-protocol analysis, a higher proportion of participants in the PRAMEV group achieved this target compared with the control group (15 of 59 [25.4%] vs 10 of 81 [12.3%]; OR, 2.52; 95% CI, 1.03-6.13; *P* = .04).

### Secondary Outcomes

No between-group differences were observed in anthropometric outcomes in the ITT analysis (Table 3), with consistent results across missing not at random analyses (eTable 1 in Supplement 2). In the per-protocol analyses, the PRAMEV group showed greater reductions in weight (−2.2 kg; adjusted mean difference, −2.4; 95% CI, −3.9 to −0.9; *P* = .002), weight percentage (−2.5%; adjusted mean difference, −2.0%; 95% CI, −3.4% to −0.6%; *P* = .005), and BMI (−1.1; adjusted mean difference, −1.1; 95% CI, −1.8 to −0.4; *P* = .002) (Table 3).

**Table 3.** Anthropometric Outcomes According to Analysis Population^a^

Outcome	Median (IQR) [No. of participants with available data]		Adjusted mean difference (95% CI)^b^	*P* value
	PRAMEV group	Control group		
**Intention-to-treat analysis**				
Weight change, kg	−2.1 (−4.5 to 0.6) [97]	−0.7 (−2.7 to 1.1) [103]	0.6 (−0.8 to 2.0)	.42
Weight change, %	−2.2 (−4.1 to 0.7) [97]	−0.7 (−2.9 to 1.3) [103]	0.6 (−0.7 to 1.9)	.37
BMI change	−0.9 (−2.1 to 0.2) [97]	−0.4 (−1.2 to 0.4) [103]	0.5 (−0.1 to 1.2)	.11
Waist circumference change, cm	−3.4 (−7.9 to 0.3) [97]	−2.1 (−5.0 to 0.8) [103]	1.6 (−0.3 to 3.4)	.10
**Per-protocol analysis**				
Weight change, kg	−2.2 (−4.6 to 0.1) [59]	−0.7 (−2.4 to 1.3) [81]	−2.4 (−3.9 to −0.9)	.002
Weight change, %	−2.5 (−5.0 to 0.1) [59]	−0.6 (−2.6 to 1.5) [81]	−2.0 (−3.4 to −0.6)	.005
BMI change	−1.1 (−2.3 to −0.1) [59]	−0.3 (−1.0 to 0.5) [81]	−1.1 (−1.8 to −0.4)	.002
Waist circumference change, cm	−3.4 (−7.9 to 0.4) [52]	−2.0 (−5.0 to 1.0) [75]	−1.2 (−3.5 to 1.1)	.30

Abbreviations: BMI, body mass index (calculated as weight in kilograms divided by height in meters squared); PRAMEV, Advanced Program for Intensive Lifestyle Modification.

^a^ The intention-to-treat analysis included all randomized participants (97 in the PRAMEV group and 103 in the control group). Missing outcome data were handled using multiple imputation by chained equations under the missing at random assumption. The per-protocol analysis was restricted to participants who completed the 20-week follow-up.

^b^ Adjusted mean differences and 95% CIs were estimated using linear regression models adjusted for baseline values, age, and sex.

The difference between current and desired weight decreased in the PRAMEV group compared with the control group (median [IQR], 2.6 [0.0 to 5.0] vs 0.0 [0.0 to 4.0] kg; *P* = .006) (Table 4), indicating a shift toward more realistic body weight expectations. The PRAMEV group showed favorable changes in ALT (median [IQR], −4.0 [−7.8 to 0.0] vs −2.0 [−4.9 to 1.9] U/L [to convert to microkatals per liter, multiply by 0.0167]; *P* = .01), HDL-C (median [IQR], +3.0 [0.1 to 4.7] vs +1.2 [−2.0 to 3.6] mg/dL [to convert to millimoles per liter, multiply by 0.0259]; *P* = .01), LDL-C (median [IQR], −5.0 [−16.4 to 7.0] vs −0.1 [−7.5 to 12.4] mg/dL [to convert to millimoles per liter, multiply by 0.0259]; *P* = .02), and triglycerides (median [IQR], −7.51 [−27.5 to 5.6] vs −2.0 [−18.0 to 15.0] mg/dL [to convert to millimoles per liter, multiply by 0.0113]; *P* = .01) (Table 4; eTable 2 in Supplement 2). Total median (IQR) energy expenditure decreased in the PRAMEV group (−2.2 [−21.0 to 9.0] kcal/d) and increased slightly in the control group (+6.0 [−10.0 to 22.0] kcal/d) (*P* = .004), whereas skeletal muscle mass decreased in the PRAMEV group (−0.0 [−0.7 to 0.4] kg) and increased in the control group (+0.2 [−0.3 to 0.6] kg) (*P* = .007) (Table 4).

**Table 4.** Between-Group Differences in Outcomes From Baseline to Week 20

Outcome	PRAMEV group^a^	Control group^a^	Between-group difference	*P* value
Desired weight, kg	2.6 (0.0 to 5.0)	0.0 (0.0 to 4.0)	2.6	.01
SBP, mm Hg	−2.3 (−6.2 to 2.3)	−2.1 (−5.9 to 3.0)	−0.2	.93
DBP, mm Hg	−1.3 (−7.0 to 7.2)	−1.0 (−11.0 to 7.2)	−0.3	.46
HR, beats/min	7.3 (3.0 to 11.4)	9.9 (3.7 to 15.0)	−2.5	.07
TEE, kcal/d	−2.2 (−21.0 to 9.0)	6.0 (−10.0 to 22.0)	−8.2	.004
SMM, kg	−0.0 (−0.7 to 0.4)	0.2 (−0.3 to 0.6)	−0.2	.007
BF, %	−0.9 (−1.8 to 0.3)	−0.7 (−2.0 to 0.1)	−0.2	.96
Physical activity energy expenditure, kcal/d	69.0 (0.0 to 190.7)	0.0 (0.0 to 170.8)	69.0	.24
AST, U/L	−1.6 (−3.5 to 1.6)	−2.0 (−4.0 to 1.0)	0.4	.59
ALT, U/L	−4.0 (−7.8 to 0.0)	−2.0 (−4.9 to 1.9)	−2.0	.01
Glucose, mg/dL	0.0 (−7.0 to 5.5)	2.0 (−6.0 to 9.0)	−2.0	.09
HbA_1c_, %	0.0 (−0.1 to 0.2)	0.1 (−0.1 to 0.2)	−0.1	.09
LDL-C, mg/dL	−5.0 (−16.4 to 7.0)	−0.1 (−7.5 to 12.4)	−4.9	.02
HDL-C, mg/dL	3.0 (0.1 to 4.7)	1.1 (−2.0 to 3.6)	1.9	.01
Serum total cholesterol, mg/dL	−2.7 (−18.0 to 11.3)	0.4 (−15.7 to 15.0)	−3.1	.29
Triglycerides, mg/dL	−7.5 (−27.5 to 5.6)	−2.0 (−18.0 to 15.0)	−5.5	.01
Stress score	−1.5 (−6.9 to 4.1)	−2.0 (−6.6 to 4.0)	0.5	.51
Depression score	−1.7 (−7.9 to 2.0)	−2.0 (−8.4 to 2.6)	0.3	.84
Anxiety score	−1.4 (−6.0 to 3.0)	−1.7 (−7.0 to 4.0)	0.3	>.99
BES score	−3.0 (−9.2 to 1.2)	−1.0 (−7.5 to 3.1)	−2.0	.08
Total energy intake, kcal	−26.8 (−204.1 to 159.2)	34.3 (−282.9 to 249.4)	−61.1	.40
Total protein, % TEI	−0.1 (−7.4 to 50.2)	−0.3 (−5.3 to 4.7)	0.2	.19
Total carbohydrate, % TEI	−8.5 (−37.9 to 6.0)	0.7 (−10.3 to 5.8)	−9.1	.13
Total lipid, % TEI	−8.0 (−20.6 to 6.4)	−1.1 (−14.7 to 8.2)	−6.9	.61
Saturated fat, % TEI	−1.6 (−8.9 to 5.6)	0.1 (−5.7 to 4.2)	−1.6	.40
Monounsaturated fat, % TEI	−1.0 (−4.8 to 10.8)	−0.8 (−6.2 to 1.9)	−0.2	.33
Polyunsaturated fat, % TEI	0.7 (−5.1 to 7.8)	−0.5 (−4.3 to 1.6)	1.1	.19
Dietary cholesterol, % TEI	20.7 (−141.1 to 190.1)	−5.4 (−109.5 to 124.7)	26.1	.89
Total fiber, g	1.2 (−2.1 to 5.6)	0.9 (−5.4 to 6.4)	0.4	.67
G1, % TEI	4.6 (−11.7 to 18.0)	2.2 (−11.5 to 20.2)	2.5	.86
G2, % TEI	0.4 (−2.2 to 2.3)	0.1 (−1.5 to 3.0)	0.3	.41
G3, % TEI	2.7 (−8.6 to 13.7)	0.0 (−9.8 to 9.2)	2.6	.38
G4, % TEI	−6.8 (−16.6 to 6.8)	−3.5 (−14.4 to 8.5)	−3.3	.92

Abbreviations: ALT, alanine aminotransferase; AST, aspartate aminotransferase; BF, body fat; BES, Binge Eating Scale; DBP, diastolic blood pressure; G1, group 1 (unprocessed or minimally processed foods); G2, group 2 (processed culinary ingredients); G3, (processed foods); G4, (ultraprocessed foods and beverages); HbA_1c_, glycated hemoglobin; HDL-C, high-density lipoprotein cholesterol; HR, heart rate; LDL-C, low-density lipoprotein cholesterol; PRAMEV, Advanced Program for Intensive Lifestyle Modification; SBP, systolic blood pressure; SMM, skeletal muscle mass; TEE, total energy expenditure.

SI conversion factors: To convert glucose to millimoles per liter, multiply by 0.0555; cholesterol to millimoles per liter, multiply by 0.0259; triglycerides to millimoles per liter, multiply by 0.0113; AST and ALT to microkatals per liter, multiply by 0.0167.

^a^ Data are presented as median (IQR) and compared using Mann-Whitney *U* test.

Psychosocial scores and dietary assessments showed no significant between-group differences in the ITT or per-protocol analyses (Table 4; eTable 3 in Supplement 2). Within-group analyses showed a decrease in median (IQR) depression scores in the control group (12.0 [6.0-24.0] to 8.0 [4.0-18.0]; *P* = .03) and a reduction in median (IQR) binge eating scores (11.0 [5.0-18.0] to 9.0 [3.0-13.0]; *P* = .01) and monounsaturated fat intake (mean [SD] of 10.3% [3.0%] to median [IQR] 7.6% [6.7%-9.2%] of total energy intake; *P* = .009) in the PRAMEV group (eTable 3 in Supplement 2).

### Exploratory Outcomes

Antiobesity medication use was similar between groups at baseline, including liraglutide, sibutramine, and orlistat, except for higher topiramate use in the PRAMEV group (62/97 [63.9%] vs 50/103 [48.5%]; *P* = .03). No differences were observed at week 20. Over time, use of at least one medication increased in controls (77 of 103 [74.8%] to 73 of 81 [90.1%]; *P* = .008), driven by topiramate (50 of 103 [48.5%] to 53 of 81 [65.4%]; *P* = .03), with no changes in the PRAMEV group (eFigure 1 in Supplement 2).

Overall follow-up completion was 70.0% (140 of 200), with no significant correlations between session attendance and clinical or psychosocial outcomes (eTable 4 in Supplement 2). The dropout rate was higher in the PRAMEV group than in the control group (38 of 97 [39.2%] vs 22 of 103 [21.4%]; *P* = .008); within the PRAMEV group, dropout was driven primarily by missed final visits (17 of 38 [44.7%]) and difficulty adhering to the protocol (11 of 38 [28.9%]) (Figure). Complementary analyses of baseline characteristics according to dropout status are provided in eTable 5 in Supplement 2. In exploratory analyses, dropout rates increased across generations, from Baby Boomers (12 of 60 [20.0%]) to Generation Z (35 of 60 [58.3%]) (*P* = .02) (eFigure 2 in Supplement 2). No adverse events or unintended effects related to the intervention were reported.

## Discussion

In this randomized clinical trial conducted within Brazil’s public health care system, no between-group difference was observed in achieving 5% or greater weight loss in the ITT analysis. To our knowledge, this was the first study to evaluate a hybrid-format, intensive lifestyle intervention in this setting. The absence of difference in the 5% or greater weight loss may reflect the modest weight change during the 20-week intervention because no between-group differences were observed in continuous weight measures.

The literature has consistently reported weight loss in lifestyle interventions.^6,14,15,16,32,33,34^ A large systematic review^6^ found a pooled mean weight loss of 2.4 kg greater than controls at 12 to 18 months. Similarly, in shorter-term interventions lasting 13 to 26 weeks, mean weight loss of 2.4 kg (95% CI, −4.44 to −0.37 kg) has been observed relative to controls in programs combining dietary and physical activity components and delivered through both remote and in-person formats.^34^

In our study, no differences were observed between groups in weight loss outcomes. However, it is important to consider that approximately 75% of participants in both groups were receiving antiobesity pharmacotherapy at baseline (eFigure 1 in Supplement 2) and had achieved prior weight loss exceeding that typically observed with these medications^15,35,36,37,38^ (Table 2). Consequently, the potential for further intervention-related weight loss may have been constrained.

In contrast to most previous studies, which evaluated lifestyle interventions as standalone treatments^12,13,14^ or when initiated concurrently with pharmacotherapy,^39,40^ our study assessed the added effect of an intensive lifestyle intervention in patients receiving ongoing, optimized medical treatment, addressing an important gap in the literature. Evidence suggests that such interventions should not be discontinued when weight loss is minimal because approaches prioritizing diet quality^41,42,43^ and physical activity^44,45,46^ rather than weight loss alone may confer meaningful cardiometabolic benefits independent of weight change.

Although no differences were observed in the ITT analysis, per-protocol analyses showed higher rates of 5% or greater weight loss and greater reductions in anthropometric outcomes in the intervention group. These findings suggest that adherence may influence intervention effectiveness, although selection bias cannot be excluded.

In this context, although no individualized caloric prescriptions were provided, the intervention may have supported additional weight loss among adherent participants. This approach enhances scalability by reducing dependence on one-on-one consultations. Prior studies have supported the efficacy of structured group programs without personalized dietary plans when adherence and behavioral reinforcement are maintained.^6,47,48^

The intervention improved metabolic parameters, reducing LDL-C, triglyceride, and ALT levels and increasing HDL-C levels. These changes may reflect a synergistic effect beyond pharmacologic therapy and support integrating behavioral strategies into obesity care, consistent with a meta-analysis^49^ showing proportional improvements in lipid profiles with weight loss.

Despite equivalent caloric intake across groups, the findings must be interpreted with caution due to the well-documented underreporting of dietary intake in obesity studies.^50,51,52,53^ Even individuals with successful weight loss histories may underestimate intake by 25% to 30%.^52^ In our sample, reported intake appeared lower than estimated total energy expenditure, even after excluding implausible values (<1000 kcal/d), urging cautious interpretation.

The predominance of female participants was consistent with prior studies^54,55^ indicating that women were more likely to engage in weight loss interventions, possibly reflecting sociocultural pressures, body dissatisfaction, and greater health-seeking.^54,56,57^ Although no differences were observed in psychosocial outcomes, the PRAMEV group had reduced desired weight, suggesting a shift in expectations. This finding may be consistent with program components addressing internalized weight stigma, promoting body acceptance, and emphasizing obesity as a chronic condition. The gap between desired and clinically meaningful weight loss is well documented and often driven by stigma, which fosters unrealistic goals and undermines long-term adherence.^7,58,59^ Supporting realistic, health-oriented goals may enhance treatment satisfaction and long-term success.

Participant retention varied across generations, a pattern observed in prior studies, with higher dropout among younger individuals.^60,61,62,63^ Although the hybrid model offers logistical advantages and scalability, challenges remained regarding long-term adherence, digital literacy, and access to technological infrastructure.

### Limitations

This study has some limitations. The main limitations include the short intervention duration, which precluded assessment of long-term sustainability, and the predominantly female and racially homogeneous sample, which may limit external validity. The high dropout rate may have introduced attrition bias. Although ITT analyses with multiple imputation by chained equations under missing at random assumptions and missing not at random sensitivity analyses yielded consistent results, bias related to missing data cannot be fully excluded.

## Conclusions

In this randomized clinical trial of adults with obesity receiving care in Brazil’s public health care system, a hybrid-format lifestyle intervention did not improve the likelihood of achieving 5% or greater weight loss in the ITT analysis in a population already receiving pharmacologic treatment for obesity. However, findings from per-protocol analyses suggest that the intervention may be effective among participants who adhere to the program. Future studies should investigate the long-term effectiveness, impact on diverse populations, and potential for greater weight loss in patients not receiving pharmacologic treatment.

## Notes

Supplement 1.
Trial Protocol

Supplement 2. **eTable 1. **Sensitivity Analyses for Anthropometric Outcomes Under MNAR Assumptions (ITT Analysis) **eTable 2. **Changes in Anthropometric, Clinical, and Laboratory Variables in the Per-Protocol Analysis **eTable 3. **Clinical, Behavioral, and Dietary Measures at Baseline and Week 20 by Treatment Group **eTable 4. **Correlation Between Session Attendance and Changes in Clinical and Psychosocial Outcomes **eTable 5. **Baseline Characteristics According to Dropout Status by Study Group **eFigure 1. **Anti-Obesity Medication Use by Group and Over Time **eFigure 2. **Study Dropout Rates According to Reason and Participant Generation Protocol of Usual Medical Care

Supplement 3.
Data Sharing Statement
