# Supplementary material for: A Hybrid Lifestyle Program and Metabolic Health in Adults With Obesity: The PRAMEV Randomized Clinical Trial
Source: JAMA Netw Open. 2026 Aug 11;9(8):e2626884. doi: 10.1001/jamanetworkopen.2026.26884 (PMC13463168; doi:10.1001/jamanetworkopen.2026.26884)
Supplement: Supplementary file 1 [file jamanetwopen-e2626884-s001.pdf]

PROTOCOL

PROGRAMA AVANÇADO PARA MUDANÇA INTENSIVA DE ESTILO DE VIDA (PRAMEV)

(The Advanced Program for Intensive Lifestyle Change)

PRAMEV Research Group

August 2025

Version 1.3

Rua Dr. Enéas de Carvalho Aguiar, 155 - 5º andar, Bloco 4B - Sala 11A.
São Paulo, SP, 05403-000, Brasil
Email: graziele.amc@gmail.com
Phone: +55 11 2661-3358

SUMMARY OF CHANGES

Version 1.1 - December 2021

- The program name was changed from *Programa de Mudança Intensiva de Estilo de Vida (PROMEV)* to *Programa Avançado para Mudança Intensiva de Estilo de Vida (PRAMEV)* throughout the document. The English translation (Advanced Program for Intensive Lifestyle Change) is used in parentheses where appropriate.
- Definition of the randomization procedure with stratification by body mass index was added.

Version 1.2 - January 2022

- Inclusion of the adherence channel, with details on the weekly messaging system for self-monitoring and behavioral follow-up.

Version 1.3 - August 2025

- Minor typographical corrections and specific revisions were made to improve clarity and ensure technical standardization throughout the document. This version also includes the complete English translation.

SUMMARY

1 RESEARCH TEAM 8
2 TIMELINE OF ACTIVITIES 9
3 INTRODUCTION 12
3.1 Context 12
3.2 Relevance 12
3.3 Previous Studies 12
4 OBJECTIVES 15
4.1 Primary Objective 15
4.2 Secondary Objectives 15
5 Study Resources 16
5.1 Facilities and Institutional Structure 16
5.2 Human Resources and Multidisciplinary Support 16
5.3 Technological and Communication Resources 16
5.4 Financial Resources 17
6 STUDY METHODS 18
6.1 Study Design 18
6.2 Sample Size Calculation 18
6.3 Informed Consent 18
6.4 Inclusion and Exclusion Criteria 18
6.5 Participant Withdrawal 20
6.6 Recruitment and Selection Process 20
6.7 Randomization 20
6.8 Study Groups 21
6.8.1 Control Group 21
6.8.2 PRAMEV Group 21
6.9 Assessments and Data Collection 21
6.9.1 Weight 21
6.9.2 Height 22
6.9.3 Body Mass Index 22
6.9.4 Waist Circumference 22
6.9.5 Blood Pressure and Heart Rate 22
6.9.6 Biochemical Parameters 22
6.9.7 Body Composition Assessment 23
6.9.8 Physical Activity and Energy Expenditure Assessment 23
6.9.9 Dietary Intake Assessment 23

6.9.10 Questionnaires 24
6.9.10.1 Binge Eating Scale 24
6.9.10.2 Depression, Anxiety and Stress Scale - DASS-21 24
7 DESCRIPTION OF THE INTERVENTION 25
7.1 Program Objectives and Principles 25
7.2 General Structure of the Program 25
7.3 Goals 26
7.3.1 Weight Loss Goal 26
7.3.2 Physical Activity Goal 27
7.4 Self-Monitoring 27
7.4.1 Weight Self-Monitoring 27
7.4.2 Physical Activity Self-Monitoring 27
7.4.3 Dietary Intake Self-Monitoring 28
7.5 Lifestyle Change Instructors 28
7.6 Support Center 28
7.6.1 Question Channel 28
7.6.2 Adherence Channel 29
7.7 Weekly Structure of the Program 30
7.7.1 In-person session 32
7.7.2 Online Sessions 34
7.7.3 Video Content 38
7.7.4 Follow-up messages 43
7.8 Educational Materials 46
7.9 Compensation for Participation 46
8 DATA ANALYSIS 47
8.1 Statistical Analysis Strategies 47
9 QUALITY MONITORING OF THE INTERVENTION 49
10 DATA SECURITY 50
11 REFERENCES 51

ABSTRACT

Obesity is a chronic and multifactorial disease with high global prevalence. It is associated with various metabolic, cardiovascular, musculoskeletal, and psychosocial complications, in addition to generating substantial costs and placing a significant burden on health systems. Therapeutic options such as pharmacological treatment and bariatric surgery have proven efficacy and show promising results in obesity management. However, access to these approaches remains limited in public health systems, especially in middle-income countries such as Brazil. Furthermore, these treatments are not effective in isolation, that is, without being combined with lifestyle changes involving improved dietary quality with caloric deficit, regular physical activity, and behavioral interventions. In this context, lifestyle-change-based interventions remain the cornerstone of obesity treatment, being essential both as a standalone strategy and in combination with more recent and effective approaches. Structured health education and behavioral modification programs are therefore essential to expand access to safe, feasible, and sustainable interventions for addressing obesity on a large scale.

In this context, the *Programa Avançado para Mudança Intensiva de Estilo de Vida (PRAMEV)* will be developed with the aim of promoting a minimum weight loss of 5% in adults with obesity through a structured, feasible, and scalable intervention. The program combines in-person and online components, constituting a hybrid approach.

The study will be conducted at the Obesity and Metabolic Syndrome Outpatient Clinic of the Hospital das Clínicas, Faculty of Medicine, University of São Paulo. Adults aged 18 years or older, with a Body Mass Index (BMI) ≥ 30 kg/m², and access to internet-connected devices (smartphone, tablet, or computer) will be included.

To reduce methodological biases, ensure sample homogeneity, allow greater control of factors influencing outcomes, and ensure participant safety, individuals with a history of bariatric surgery, pregnant or breastfeeding women, illiterate individuals, patients with endocrine disorders such as uncompensated hypothyroidism, genetic syndromes associated with obesity, cognitive impairment, or uncontrolled psychiatric disorders will be excluded. Eligible participants will be recruited between January and March 2022 and must already be receiving clinical follow-up at the unit.

This is a randomized clinical trial. Eligible participants will be randomly assigned, in a 1:1 ratio, to either the intervention group (PRAMEV) or the control group, through stratified randomization by BMI to ensure group homogeneity. All participants will be followed for 20 weeks.

The control group will follow standard medical care only, with in-person assessments scheduled at baseline (week 0) and at the end of the intervention (week 20). The PRAMEV group will undergo the same clinical follow-up and, additionally, will participate in a structured intervention consisting of two in-person

group sessions, four online sessions via the Google Meet platform, and the weekly delivery of videos and follow-up messages through the WhatsApp application. Throughout the 20-week intervention, PRAMEV participants will also have access to a support center, where a designated professional will provide guidance and answer questions upon request, aiming to foster rapport and personalize support, with a focus on adherence.

The PRAMEV intervention will incorporate six key components: (1) group sessions to encourage peer support and experience sharing; (2) frequent contact with participants; (3) individualized support through the support center; (4) standardized sessions to teach behavioral strategies focused on weight loss and physical activity promotion; (5) adaptation of educational materials to provide information and reinforce strategies; (6) continuous support provided by lifestyle instructors.

Data collection will be carried out for both groups at weeks 0 and 20, including measurements of weight, height, body mass index, waist circumference, blood pressure, heart rate, laboratory tests (lipid profile, liver enzymes, blood glucose, and glycated hemoglobin), body composition assessment via bioelectrical impedance, and the administration of validated questionnaires: Binge Eating Scale, Depression, Anxiety and Stress Scale, and Food Record. All measurements will follow standardized clinical protocols and will be conducted by nutritionists or supervised interns.

LIST OF ABBREVIATIONS

DASS-21	Depression, Anxiety and Stress Scale
DPP	Diabetes Prevention Program
BES	Binge Eating Scale
HCFMUSP	Clinics Hospital of the University of São Paulo Medical School
IGOBE	Group Intervention in OBEsity
Look AHEAD	Action for Health in Diabetes
MET	Metabolic Equivalent of Task
PRAMEV	The Advanced Program for Intensive Lifestyle Change
PREDIMED-Plus	PREvención con DIeta MEDiterránea

1 RESEARCH TEAM

Name	Role/Contact	DDA	R	AQI	DA
Graziele Souza de Menezes Amorim Coelho	Coordinator<br>graziele.amc@gmail.com	Y	Y	Y	Y
Renata Bressan Pepe	Co-Investigator and Research Analyst<br>nutrirenatabressan@gmail.com	Y	N	Y	Y
Ariana Fernandes	Co-Investigator and Statistician<br>arianaefernandes@gmail.com	Y	N	Y	Y
Patrícia Aparecida Cruz	Co-Investigator<br>paty.nutri1@hotmail.com	Y	N	Y	Y
Andrea Samara Audi	Co-Investigator<br>andreaaudi1@gmail.com	Y	N	N	N
Isabelle Cristine Boromello	Research Assistant<br>isa.boromello@gmail.com	N	Y	N	N
Victoria Tsubota Manrique	Research Assistant<br>victoriamanrique@outlook.com	N	Y	N	N
Cintia Cercato	Co-Investigator<br>ccercato@netpoint.com.br	Y	N	N	Y
Marcio Corrêa Mancini	Supervisor<br>mmancini@usp.br	Y	N	N	Y
Maria Edna de Melo	Principal Investigator (key), Supervisor and Statistician<br>medna@usp.br	Y	N	N	Y

DDA: Database Access; R: Recruitment; AQI: Application of Questionnaires and Interviews; DA: Data Analysis; Y: Yes; N: No.

2 TIMELINE OF ACTIVITIES

The Activity Schedule described below presents the timeline of the study phases, organized by methodological categories. The activities are grouped by domain and distributed throughout the protocol weeks, including the pre-intervention phase (week -12) and the follow-up period (weeks 0 to 20).

**Week**	**-12**	**0**	**1**	**2**	**3**	**4**	**5**	**6**	**7**	**8**	**9**	**10**	**11**	**12**	**13**	**14**	**15**	**16**	**17**	**18**	**19**	**20**
Documentation and Consent																						
Response to Participation Invitation	x																					
	The invitation should be made approximately three months prior to the routine appointment already scheduled with the endocrinology team, either by phone call or via WhatsApp message.																					
ICF		x																				
	The consent form must be presented in person by a member of the team, along with an explanation of the study objectives, procedures, and participant rights. Any questions should be clarified before signing.																					
Demographic data																						
Demographic data will be collected in advance through the system, at the moment the patient agrees to participate in the screening. On the day of the screening, this information will be reviewed and supplemented, if necessary.																						
Age	x																					
Gender	x																					
Ethnicity	x																					
Education level	x																					
Marital status	x																					
Monthly household income	x																					
Clinical assessment																						

Week	-12	0	1	2	3	4	5	6	7	8	9	10	11	12	13	14	15	16	17	18	19	20
Blood pressure		x																				x
Heart rate		x																				x
Complementary anamnesis		x																				x
	The complementary anamnesis aims to reinforce and complement the data collected through the system by gathering information on weight gain history, medication use, comorbidities, family history, lifestyle habits, and dietary patterns.																					
Physical assessment																						
Weight		x																				x
Height		x																				
Waist circumference		x																				x
Bioelectrical impedance		x																				x
	Weight will initially be measured using a platform scale for the screening process. If the participant is deemed eligible according to the inclusion criteria, they will undergo bioelectrical impedance analysis, and the weight obtained from this assessment will be used as the reference for the study data analysis.																					
Laboratory assessments																						
Laboratory data from week 0 (baseline) will be collected through the system, based on tests previously requested by the endocrinology team. Only tests performed within six months prior to the start of the intervention will be included. The tests for week 20 (post-intervention) will be requested directly by the PRAMEV team. All sample collections will be carried out at the outpatient clinic laboratory, following institutional protocols, including the requirement of fasting according to standard guidelines.																						
Blood glucose		x																				x
Glycated hemoglobin		x																				x
Total cholesterol		x																				x
LDL-C		x																				x

**Week**	**-12**	**0**	**1**	**2**	**3**	**4**	**5**	**6**	**7**	**8**	**9**	**10**	**11**	**12**	**13**	**14**	**15**	**16**	**17**	**18**	**19**	**20**
Non-HDL-C		x																				x
HDL-C		x																				x
Triglycerides		x																				x
AST		x																				x
ALT		x																				x
GGT		x																				x
Assessment of Psychosocial and Nutritional Aspects																						
DASS-21		x																				x
BES		x																				x
DIR		x																				x
Intervention																						
In-person session		x																				x
Online session						x				x				x			x					x
Video				x				x				x				x				x		
Follow-up message			x		x		x		x		x		x		x		x		x		x	
Program Adherence			x	x	x	x	x	x	x	x	x	x	x	x	x	x	x	x	x	x	x	
Self-monitoring			x	x	x	x	x	x	x	x	x	x	x	x	x	x	x	x	x	x	x	

ICF: Informed Consent Form; LDL-C: Low-Density Lipoprotein Cholesterol; Non-HDL-C: Non-High-Density Lipoprotein Cholesterol; HDL-C: High-Density Lipoprotein Cholesterol; AST: Aspartate Aminotransferase; ALT: Alanine Aminotransferase; GGT: Gamma-Glutamyl Transferase; DASS-21: Depression, Anxiety, and Stress Scale; BES: Binge Eating Scale; DIR: Dietary Intake Records.

3 INTRODUCTION

3.1 Context

Obesity is a chronic disease with high prevalence, characterized by excessive accumulation of body fat and strongly associated with a range of metabolic, cardiovascular, and psychosocial comorbidities(1-3). Its etiology is complex and involves genetic, environmental, behavioral, and social factors, making its management a significant challenge(4). In Brazil, the situation is equally concerning: in 2019, more than 60% of the Brazilian population was overweight, and 25.9% was classified as obese(5).

Pharmacological and surgical interventions are important strategies in the treatment of obesity, especially in more severe cases(6, 7). However, access to these treatments is limited within the public health system and is often restricted to specialized referral centers(8). In this context, lifestyle modification remains the cornerstone of therapy and the most viable alternative for the majority of the population, being essential even when combined with more complex interventions(9-12). Therefore, structured lifestyle change programs are crucial for expanding the reach of care and offering practical and effective alternatives for addressing obesity.

3.2 Relevance

Several national and international clinical guidelines recognize that lifestyle-based interventions, which include healthy eating with a caloric deficit, regular physical activity, and behavioral strategies, are fundamental for weight control and the prevention of obesity-related diseases(13). In addition to promoting metabolic and functional benefits, these interventions have a favorable safety profile and are recommended as the foundation for treating obesity and its complications.

However, large-scale implementation remains a challenge, particularly in public health contexts that face structural, economic, and logistical limitations(14). Developing accessible, standardized, and evidence-based programs adapted to local realities is a promising strategy to improve obesity management in public health systems(15). The adoption of hybrid interventions, combining in-person sessions with online resources, expands reach, reduces operational costs, and facilitates patient adherence.

3.3 Previous Studies

The concept of intensive lifestyle therapy was initially developed and widely established by the Diabetes Prevention Program (DPP), a large-scale, multicenter clinical trial conducted in the United States. The main objective of the DPP was to evaluate whether an intensive lifestyle intervention or the use of

metformin would be effective in preventing or delaying the onset of type 2 diabetes mellitus in individuals with prediabetes, that is, those at high risk for the disease(16).

The structured intervention developed by the DPP laid the foundational pillars of this model, characterized by a set of integrated strategies: nutritional guidance focused on caloric reduction, regular physical activity (at least 150 minutes per week), cognitive behavioral therapy techniques, and continuous participant engagement throughout the intervention, with particular emphasis during the first six months. The study's 3,234 participants were randomized and monitored with the goal of achieving a 7% reduction in initial body weight within the first 24 weeks. To that end, they were instructed to maintain a daily caloric deficit of 500 to 1,000 kcal, to use energy-controlled meal replacements, and to incorporate self-monitoring tools and cognitive restructuring strategies into their daily routines(17).

At the end of the follow-up period, there was a 58% reduction in the incidence of type 2 diabetes mellitus in the intensive lifestyle intervention group, compared to a 31% reduction in the group treated with metformin, over an average follow-up of 2.8 years (Figure 1). Participants in the intensive intervention group also became more physically active and achieved significantly greater weight loss than those assigned to receive metformin or placebo (Figure 2), demonstrating that intensive lifestyle modification is an effective strategy for overall health improvement.(18).

Figure 1: Cumulative incidence of diabetes in the study groups

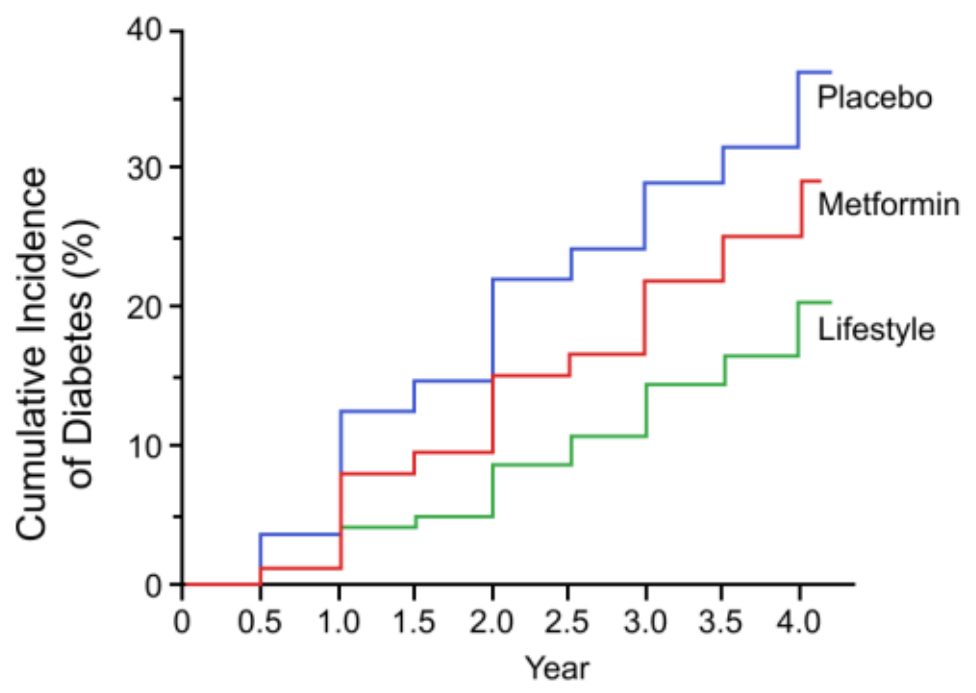

Diagnosis based on the criteria of the American Diabetes Association.

Figure 2: Changes in body weight and physical activity

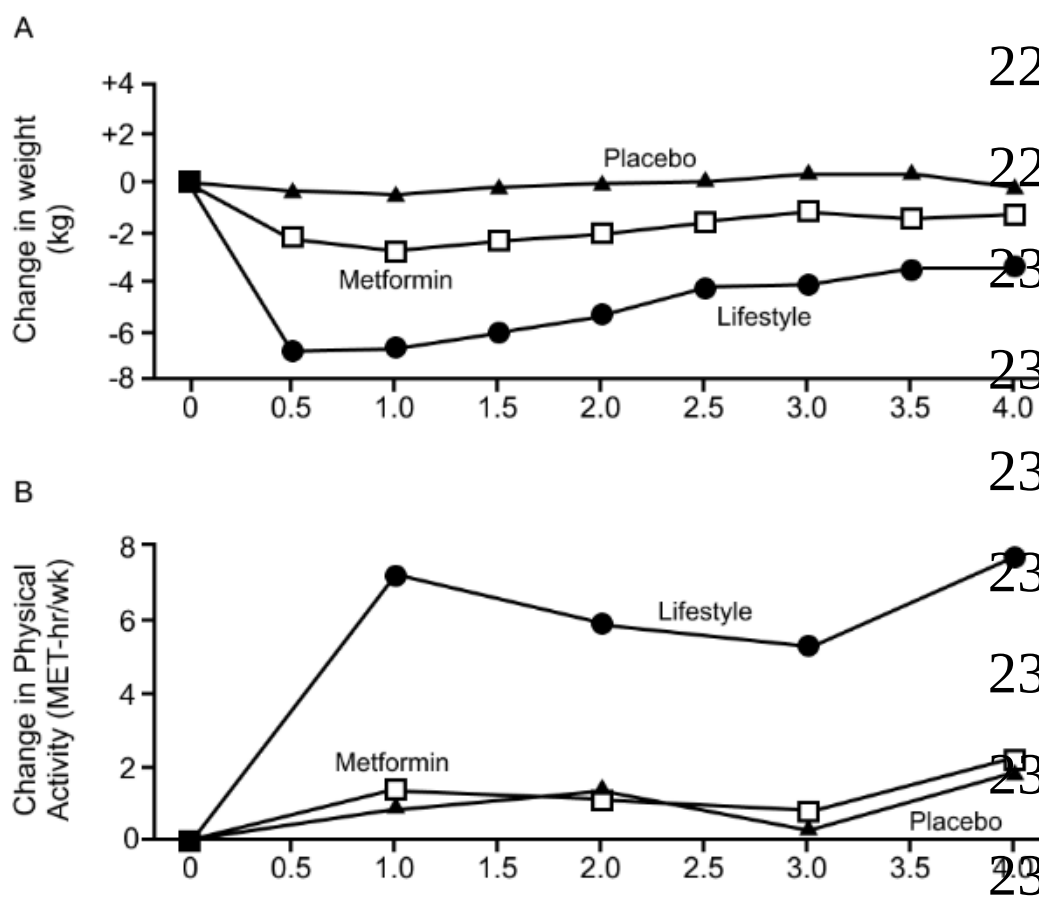

Changes in body weight (Graph A) and physical activity levels (Graph B), according to the study groups.

Inspired by this pioneering model, several adaptations have emerged, including Look AHEAD (Action for Health in Diabetes), focused on individuals with type 2 diabetes mellitus(9); PREDIMED-Plus (PREvención con DIeta MEDiterránea), emphasizing the Mediterranean diet and lifestyle in adults with metabolic syndrome(19); and IGOBE (Group Intervention in OBEsity), which implemented group-based interventions to promote behavioral change in patients with severe obesity(20). These models have demonstrated that structured strategies with clear weight loss goals, group education, and continuous support are effective in reducing risk factors associated with weight gain.

PRAMEV was developed based on well-established intensive lifestyle change interventions, incorporating key elements that promote adherence and sustainable outcomes. The program was adapted to

ensure feasibility within the Brazilian context, taking into account the cultural, social, and structural characteristics of the population. It is a hybrid intervention, consisting of in-person and online group sessions, as well as continuous remote follow-up, which enhances its applicability in public health services. Table 1 provides a summary of the main interventions, in addition to the DPP, that served as the foundation for the structure of PRAMEV.

Table 1 - Interventions Based on Lifestyle Change Programs

**Programs**	**Objective**	**Methods**	**Results**
Look AHEAD(21)	Evaluate the impact of weight loss on cardiovascular outcomes in type 2 diabetes mellitus	CG: Standard health education<br>IG: Intensive intervention with caloric deficit, meal replacements, ≥175 min/week of physical activity, and weekly behavioral sessions	4-year follow-up:<br>CG: 1.1% weight loss<br>IG: 4.7% weight loss<br>Improvements in BP, FPG, and lipid profile
PREDIMED-Plus(19)	Evaluate ↓ weight loss in the prevention of cardiovascular events	CG: Mediterranean diet<br>IG: Monthly group and individual sessions – Mediterranean diet (-30% kcal), physical activity, weight loss goals, and behavioral counseling	12-month follow-up:<br>CG: -0.7 kg; 11.9% of participants lost ≥5%<br>IG: -3.2 kg; 33.7% of participants lost ≥5%<br>CVD risk: ↓ WC, FPG, TG and ↑ HDL
IGOBE(20)	Evaluate ↓ weight loss and lifestyle habits	CG: Standard treatment with endocrinologist and nurse<br>IG: Weekly group sessions for 6 weeks + 2 follow-up visits at 6 and 12 months (diet, physical activity, and behavioral strategies)	12-month follow-up:<br>CG: +3,0%<br>IG: -7,1%

CG: Control Group; **IG:** Intervention Group; **BP:** Blood Pressure; **FPG:** Fasting Plasma Glucose; **CVD risk:** Cardiovascular Disease Risk; **WC:** Waist Circumference; **TG:** Triglycerides; **HDL:** High-Density Lipoprotein.

4 OBJECTIVES

4.1 Primary Objective

To evaluate whether an intensive lifestyle change program, delivered in a hybrid format and combined with standard medical care, leads to greater weight loss in adults with obesity compared to standard medical care only.

4.2 Secondary Objectives

To evaluate the effect of an intensive lifestyle change program, delivered in a hybrid format, on the following parameters:

- Anthropometric and body composition indicators: body mass index, waist circumference, fat mass, and skeletal muscle mass;
- Biochemical parameters: fasting glucose, glycated hemoglobin, total cholesterol and fractions, triglycerides, and liver enzymes;
- Dietary intake;
- Physical activity;
- Psychosocial variables, including body image perception, and symptoms of depression, stress, and anxiety;
- Binge eating behavior.

5 STUDY RESOURCES

5.1 Facilities and Institutional Structure

The study will be conducted at the Obesity and Metabolic Syndrome Outpatient Clinic of the Division of Endocrinology and Metabolism at HCFMUSP. This is a referral center for specialized care of patients with obesity, integrated into a large academic institution, with adequate infrastructure for the development of clinical studies, including anthropometric and laboratory data collection and the implementation of intervention protocols.

5.2 Human Resources and Multidisciplinary Support

The team responsible for conducting the study will consist of nutritionists, endocrinologists, and nutrition undergraduate interns, all trained to carry out screening procedures, intervention delivery, clinical follow-up, data collection, and participant support. The joint work of these professionals will ensure the multidisciplinary approach necessary for the comprehensive management of patients with obesity throughout the study.

In addition, the hospital's own institutional resources will be used for the performance of laboratory tests and bioelectrical impedance analysis, which are already part of the outpatient clinic's routine care. This structure will contribute to the standardization and reliability of the clinical and metabolic measurements to be collected during the study.

5.3 Technological and Communication Resources

To enable the hybrid format of the intervention, accessible and widely available digital platforms will be used. Online group sessions will be conducted via the Google Meet platform, while the delivery of videos, goals, and reinforcement messages will occur through the WhatsApp application.

The choice of digital tools is feasible due to the widespread access to mobile phones and the internet in Brazil. According to the 2019 National Household Sample Survey, published by the Brazilian Institute of Geography and Statistics, 94.0% of Brazilian households owned a mobile phone, and 82.7% had internet access. Among connected households, 99.5% used mobile phones as their main means of accessing the internet(22). These data highlight the extensive use of mobile technology in the country, even before the COVID-19 pandemic, and support the use of digital tools as viable, practical, and low-cost resources for the remote and continuous follow-up of participants in the intervention.

5.4 Financial Resources

This study will not receive external public or private funding. All planning and implementation of the intervention will be carried out by volunteer professionals, the research team, and the local staff of the Obesity and Metabolic Syndrome Outpatient Clinic at HCFMUSP.

The resources used during the study will already be part of the clinic's routine care, and no additional investments will be required. Laboratory tests and other clinical assessments, as part of standard medical care, will be used for research data collection purposes. Similarly, the physical and technological infrastructure of the service will be used according to its regular availability, ensuring the ethical and operational feasibility of the study without generating additional costs.

6 STUDY METHODS

6.1 Study Design

This study will be conducted as a randomized clinical trial with a duration of 20 weeks. Eligible participants will be allocated in a 1:1 ratio to either the PRAMEV group or the control group through stratified randomization by body mass index, in order to ensure homogeneity between groups. The PRAMEV group's hybrid intervention will consist of in-person and online group sessions, continuous remote follow-up, and standard medical care.

6.2 Sample Size Calculation

The sample size calculation was based on a study that assessed the effectiveness of the methodology employed by the DPP(15) in a real-world setting, in which the mean weight loss in the intervention group was approximately 5 kg (standard deviation of 5 kg). Assuming a smaller expected weight loss in the control group, estimated at around 2 kg (standard deviation of 2 kg), a sample size of 27 participants per group was estimated to be sufficient to detect significant differences between groups with 80% statistical power and a 5% significance level (two-tailed test).

However, considering the high dropout rate commonly observed in obesity treatment programs (estimated at up to 30%) and the fact that the proposed intervention is based on a previously validated, effective, and safe methodology, the sample size will be pragmatically expanded to include all patients with scheduled appointments at the Obesity and Metabolic Syndrome Outpatient Clinic of HCFMUSP during the recruitment period (January to March 2022), provided they meet the eligibility criteria. The final estimated sample size is approximately 100 participants per group.

6.3 Informed Consent

The informed consent process was conducted by members of the research team, who provided detailed explanations about the study, its objectives, risks, and benefits. Participants who agreed to take part signed the informed consent form prior to the beginning of data collection.

6.4 Inclusion and Exclusion Criteria

Eligibility criteria were defined to ensure participant safety, reduce methodological biases, maintain sample homogeneity, and allow greater control over factors influencing study outcomes.

Table 2 - Inclusion and Exclusion Criteria

Inclusion Criteria:

- Age 18 years or older
- Body mass index (BMI) ≥ 30 kg/m²
- Access to devices for remote care (smartphone, computer, or tablet).

Exclusion Criteria:

- Non-adherence to the study protocol
- Pregnant women
- Breastfeeding women
- History of bariatric surgery
- Illiterate individuals
- Presence of decompensated endocrine disorders
- Genetic syndromes associated with obesity
- Cognitive impairment
- Uncontrolled psychiatric disorders.

Although the study aims to develop an accessible and scalable intervention for managing obesity within the public health system, certain exclusion criteria will be applied to ensure methodological feasibility and the integrity of the data to be collected. Participants without access to internet-connected devices will be excluded, as the proposed intervention depends on the use of digital platforms (Google Meet and WhatsApp).

Patients with cognitive impairment, genetic syndromes associated with obesity, or low literacy will not be denied care through PRAMEV. However, their formal participation in the research will not be feasible, as in most cases they will be unable to interpret the questionnaires or complete the required tasks.

It is acknowledged that this limitation may restrict the representativeness of the sample, particularly among populations experiencing greater social vulnerability. Nevertheless, this decision aims to preserve the standardization of the intervention, reduce loss to follow-up, and ensure the quality of the data collected.

In the future, PRAMEV will be adapted into more inclusive versions, incorporating in-person or phone-based support, assistance from community health workers, and the use of simplified platforms. These adaptations will allow the program to reach individuals with lower levels of digital or formal literacy while maintaining the core structure and principles of the intervention.

6.5 Participant Withdrawal

Participation in PRAMEV is voluntary, and participants may withdraw from the study at any time without any harm to their care or their relationship with the health care unit.

No compulsory exclusion of participants is anticipated. However, if situations arise such as:

- Pregnancy;
- Physical or psychological complications that prevent continuation of the intervention;
- Medical advice against continued participation in the study.

Data collection for the research database will be discontinued from that point onward. Nevertheless, the participant may, if desired and if deemed appropriate by the team, continue to receive guidance related to the intervention, in accordance with the reasons that led to the interruption of formal follow-up.

If the participant requests full withdrawal from the study, their decision will be fully respected. In this case, no further data will be collected, but data recorded up to the date of withdrawal may still be used.

6.6 Recruitment and Selection Process

Between January and March 2022, all patients receiving care at the Obesity Outpatient Clinic of HCFMUSP will be invited to participate. Initial contact will be made via phone call or WhatsApp message, prior to the appointment with the endocrinology team. Patients who express interest will be referred, immediately after the consultation, to the screening room. Following assessment based on the inclusion and exclusion criteria, eligible and interested patients will be invited to participate in the study.

They will then proceed to the next stage of the process, which includes signing the informed consent form, completing validated questionnaires, recording dietary intake, and undergoing randomization. If the participant is allocated to the PRAMEV group, they will be immediately referred to the intervention room, where all participants scheduled for that day will begin the first group session.

6.7 Randomization

Participant allocation will be performed using the RAND function in Microsoft Excel®, with stratified randomization based on body mass index. This procedure aims to ensure balanced distribution between the control and intervention groups.

Due to the nature of the intervention, participant blinding regarding group allocation will not be possible, which may introduce performance and expectancy bias. To mitigate this effect, primary and secondary outcomes will be based on objective and measurable variables (such as body weight, biochemical parameters, and body composition assessed by bioelectrical impedance), and data will be collected by trained

assessors following standardized protocols. In addition, the psychosocial instruments to be used are validated for self-administration, which minimizes the influence of the research team. This aspect will be taken into consideration when interpreting the results.

6.8 Study Groups

6.8.1 Control Group

Participants in the control group will continue to receive standard medical care during routine consultations with the endocrinology team, during which they will receive basic lifestyle guidance. They will undergo two in-person assessments: one at week 0 (baseline) and another at week 20 (end of the intervention).

6.8.2 PRAMEV Group

Participants in the PRAMEV group, in addition to standard medical care, will take part in a structured 20-week program that includes two in-person group sessions, one at week 0 (baseline) and another at week 20 (end of the intervention), designed for clinical assessments and the delivery of in-person group sessions. The program will also include online sessions via Google Meet and the regular delivery of videos and follow-up messages via WhatsApp, ensuring weekly contact with participants throughout the intervention period.

6.9 Assessments and Data Collection

Laboratory data will be collected from tests routinely performed at the outpatient clinic. Information regarding medication use and comorbidities will be extracted from electronic medical records. Data obtained through the system will be complemented with medical history when necessary. Anthropometric assessment will be conducted by a member of the team, either a nutritionist or a nutrition intern, always under the supervision of a nutritionist.

6.9.1 Weight

Weight will be measured using a platform scale (Filizola® brand), with 100-gram divisions. The patient will be positioned with their back to the scale's display, standing upright, barefoot, wearing light clothing, centered on the equipment, with body weight evenly distributed on both feet and arms extended alongside the body, remaining still until the measurement is complete (23).

6.9.2 Height

Height, in meters, will be measured with the patient standing barefoot, upright, with straight legs and heels together, weight evenly distributed between both legs, toes slightly apart, and arms extended alongside the body. The heels, buttocks, and shoulders must touch the wall, and the head should be aligned according to the Frankfurt plane. The observer will stand to the side of the participant and use a stadiometer with a ruler to perform the measurement (23).

6.9.3 Body Mass Index

Body mass index (BMI) will be calculated by dividing the patient's weight (in kilograms) by their height (in meters) squared. The interpretation will follow the reference values for BMI classification in adults (24).

6.9.4 Waist Circumference

Waist circumference will be measured with the patient standing, arms alongside the body, feet close together, and weight evenly distributed between both feet, at the end of expiration. A non-stretchable measuring tape will be used, positioned at the midpoint between the last fixed rib (tenth) and the top of the iliac crest, keeping the tape parallel to the floor (24).

6.9.5 Blood Pressure and Heart Rate

Blood pressure will be measured using an aneroid sphygmomanometer, with the participant seated at rest for five minutes in a calm environment, back supported, and arm fully exposed and resting on a surface at heart level. A cuff of appropriate size (approximately 40% of the arm circumference) will be used, positioned midway between the olecranon and the acromion (25).

Heart rate will be measured after blood pressure assessment, using a digital pulse oximeter.

6.9.6 Biochemical Parameters

Patients will undergo routine clinical and laboratory evaluations as part of the standard protocol of the Obesity Outpatient Clinic. The following laboratory parameters will be considered: total cholesterol and its fractions (high-density lipoprotein, low-density lipoprotein, and non-HDL cholesterol), triglycerides, liver enzymes (alanine aminotransferase, aspartate aminotransferase, and gamma-glutamyl transferase), fasting glucose, and glycated hemoglobin. All participants will receive prior instructions for appropriate preparation before laboratory testing.

6.9.7 Body Composition Assessment

Body composition will be assessed using bioelectrical impedance analysis with the InBody 720 device (Biospace Co. Ltd., Seoul, Korea), a direct segmental multifrequency body composition analyzer. The device operates with a tetrapolar eight-electrode system and applies electrical currents at frequencies of 1, 5, 50, 250, 500, and 1000 kHz.

Bioelectrical impedance will also be used to estimate participants' basal metabolic rate.

6.9.8 Physical Activity and Energy Expenditure Assessment

Physical activity will be assessed based on self-reported information collected during the in-person assessments at weeks 0 and 20. The type and duration of each activity will be recorded. Activity intensity will be estimated using the Metabolic Equivalent of Task (MET), classified as light (1.6 to 2.9 METs)(26), moderate (3 to 5.9 METs), or vigorous (above 6 METs)(27). Physical activity energy expenditure will be calculated using the formula: MET × body weight (in kilograms) × duration of activity (in hours)(28).

6.9.9 Dietary Intake Assessment

Participants' dietary intake will be assessed using a two-day food record—one weekday and one weekend day—selected by the participants and collected during in-person visits at weeks 0 and 20. For the food record, participants will receive detailed verbal instructions, along with printed material containing explanations and examples. The food record is a dietary assessment method in which, after specific guidance, participants document all foods and beverages consumed, along with their respective quantities, using household measures to estimate portion sizes (29).

Dietary assessment will be based on the NOVA classification. This will include information from product labels for industrialized foods and detailed descriptions of ingredients, quantities, and preparation methods for homemade recipes. Foods will be classified into four groups: (1) unprocessed or minimally processed foods; (2) processed culinary ingredients; (3) processed foods; (4) ultra-processed foods and beverages (30).

After completing the food record, the research team will review entries for missing or incomplete information. If needed, additional details will be requested from the participant before releasing the form, and any necessary corrections will be made during the screening. Intake calculations will be performed using the Dietbox® software, which will estimate both total energy intake and the percentage distribution of macronutrients (carbohydrates, proteins, and lipids). The data will then be transferred to an Excel® spreadsheet to classify foods according to the NOVA methodology. Energy intake from each group will be calculated in absolute kilocalories and as a percentage of total daily energy intake.

6.9.10 Questionnaires

6.9.10.1 Binge Eating Scale

Screening for binge eating behavior will be conducted using the Binge Eating Scale (BES), validated in Portuguese as the "Escala de Compulsão Alimentar Periódica" (ECAP) (31). This instrument allows individuals to be classified as non-binge eaters, moderate binge eaters, or severe binge eaters. The scale consists of 16 items and 62 statements addressing behavioral manifestations, feelings, and cognitions related to the disorder. Each statement is scored from 0 (absence of binge eating) to 3 (maximum intensity), and the final score corresponds to the sum of the points assigned. Individuals scoring 17 or below will be classified as non-binge eaters; those scoring between 18 and 26 as moderate binge eaters; and those with scores of 27 or higher as severe binge eaters (32).

6.9.10.2 Depression, Anxiety and Stress Scale - DASS-21

Symptoms of depression, anxiety, and stress will be assessed using the self-administered Depression, Anxiety and Stress Scale – DASS-21, validated for the Portuguese language. The instrument consists of 21 items: seven related to depressive symptoms, seven to anxiety symptoms, and seven to stress symptoms, referring to the past seven days. Each item is rated on a scale from 0 to 3, and the score for each subscale will be calculated by summing the corresponding items and multiplying the total by 2 (33). Score classifications are presented in Table 3.

Table 3 - DASS-21 Scale

	Depression	Anxiety	Stress
Normal	0-9	0-7	0-14
Mild	10-13	8-9	15-18
Moderate	14-20	10-14	19-25
Severe	21-27	15-19	26-33
Extremely severe	28+	20+	34+

Source: Lovibond SH and Lovibond PF, 1995.

7 DESCRIPTION OF THE INTERVENTION

7.1 Program Objectives and Principles

The main objective of the program is to achieve a 5% reduction in body weight relative to the initial value. To reach this goal, an intensive lifestyle change program, PRAMEV, will be implemented, designed and refined based on the publicly available guidelines of the Diabetes Prevention Program (DPP)(17). During the adaptation of the model, the diversity of the Brazilian population and its cultural and socioeconomic characteristics will be taken into account, motivating context-specific adjustments.

To achieve the proposed goal, the program will incorporate six key elements:

1. Group sessions to promote experience sharing;
2. Frequent contact with participants;
3. Individualized support through a dedicated support center to improve program adherence;
4. Standardized sessions to teach behavioral strategies focused on weight loss and physical activity promotion;
5. Adaptation of educational materials to provide information and reinforce recommended strategies;
6. Continuous support provided by lifestyle instructors.

7.2 General Structure of the Program

In the original DPP structure, all sessions were conducted in person. The first stage included 16 predefined-content sessions, distributed over the initial 24 weeks of the intervention (17). However, considering the economic and mobility limitations faced by populations served in public health settings, as well as the impacts and restrictions associated with the COVID-19 pandemic, PRAMEV will be adapted into a hybrid format. This adaptation will allow approximately 90% of the activities to be delivered online, with only 10% conducted in person (limited to the first and last weeks of the intervention).

PRAMEV will last a total of 20 weeks, with activities organized in a cyclical sequence. The intervention will begin with an in-person group session during which participants will receive orientation about the program, including goals, participation rules, and printed materials. This will be followed by a weekly cycle consisting of: follow-up message, video, a new message, and another group session. This cycle will be repeated until week 20, when the second in-person session will take place, focused on final assessments and motivation to sustain behavior changes.

Figure 1 – Intervention Flowchart

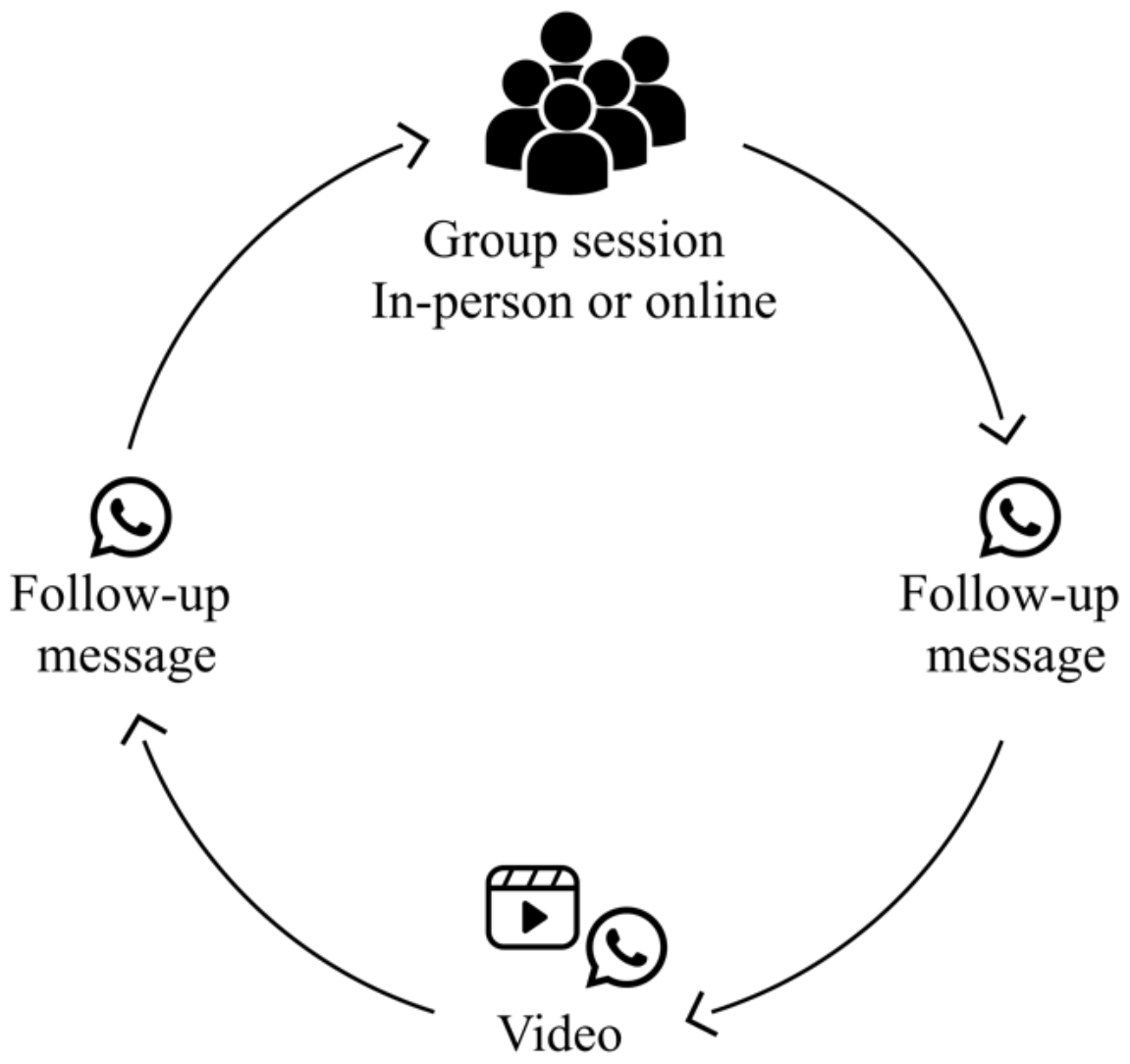

7.3 Goals

PRAMEV was developed based on clear and measurable goals, recognizing that setting specific objectives increases participant engagement and contributes to improved clinical outcomes. The goals will be grounded in scientific evidence and adapted to the capabilities of the target population, taking into account clinical, behavioral, and socioeconomic factors.

The goals will be presented during the first in-person session and reinforced throughout the 20 weeks via the program's communication channels. They will encompass two central pillars of lifestyle change: weight loss and regular physical activity. Both goals will be approached realistically, focusing on individual progress, sustainability, and the promotion of health benefits.

The two goals are detailed below.

7.3.1 Weight Loss Goal

The weight loss goal established for all PRAMEV participants will be a 5% reduction in body weight relative to their initial weight. This goal may be exceeded if the participant desires, with individual evaluation and support provided by the instructors through the support center.

During the first session, participants will be informed that a 5% weight loss is already associated with meaningful health benefits, such as improved quality of life and reduced metabolic risk factors. It will be emphasized that the goal of the intervention is not necessarily to reach a "normal" weight range, but rather to promote sustainable clinical and functional improvements.

This goal is based on both the duration of the program and evidence from the scientific literature. Studies have shown that modest weight loss of 3% to 5%, when maintained, is associated with clinically significant reductions in markers such as triglycerides, blood glucose, glycated hemoglobin, blood pressure, and the risk of developing type 2 diabetes mellitus (18, 34-38).

In light of this evidence, the 5% goal is considered appropriate, realistic, and well-supported for application in lifestyle change programs such as PRAMEV.

7.3.2 Physical Activity Goal

Participants in the PRAMEV group will be encouraged to engage in at least 150 minutes of physical activity per week, at moderate to vigorous intensity, depending on their clinical conditions and individual limitations. They will also be encouraged to incorporate more movement into their daily routines, such as taking the stairs or walking short distances, always respecting their abilities and medical guidance.

The physical activity goal follows the parameters established by lifestyle intervention programs(17) and will be reiterated throughout the program, as recommended by the World Health Organization, which advises adults to engage in at least 150 minutes of moderate-intensity physical activity per week (39).

7.4 Self-Monitoring

Self-monitoring will be a core strategy of PRAMEV to encourage behavior change related to eating habits, physical activity, and weight management. Each participant will receive a logbook with space to record food intake, physical activity, and body weight.

7.4.1 Weight Self-Monitoring

Regular self-weighing will be encouraged as a strategy to promote self-awareness and track progress throughout the program. Participants with a scale at home will be instructed to weigh themselves every other day or at least once a week, always at the same time and under similar conditions, preferably in the morning, after emptying the bladder, before breakfast, and wearing light clothing. Those without access to a home scale will be encouraged to monitor their weight weekly at nearby pharmacies. Weight will be recorded in the logbook to help identify patterns and support adjustments to strategies over time.

7.4.2 Physical Activity Self-Monitoring

Participants will be encouraged to record the type, duration, and intensity of their physical activity in the logbook. At the end of each week, they will be invited to review their entries and reflect on whether they

met the weekly goal of 150 minutes. If they reach the goal, they will be encouraged to enhance elements such as frequency or intensity. If the goal is not achieved, the team will work to identify the barriers encountered and propose feasible alternatives for the following week. Participants may request individualized support from lifestyle instructors via the support center if needed.

7.4.3 Dietary Intake Self-Monitoring

Food intake logging will be promoted as a tool for self-awareness and dietary behavior change. Participants will be instructed to record, throughout the day, the foods consumed at each meal, including portion sizes and timing. At the end of the day, they will review their entries, reflect on their choices, and compare them to the program's goals and guidelines. This practice will enable monitoring of dietary quality, identification of patterns that hinder adherence, and foster continuous self-evaluation. When necessary, the dietary records may also serve as the basis for evaluation and guidance provided by the lifestyle instructors.

7.5 Lifestyle Change Instructors

The lifestyle change instructors will play a key role in providing support and guidance to participants throughout the entire intervention. Contact with these professionals will be ongoing during the 20 weeks of the program, through WhatsApp messages and group sessions.

To strengthen rapport, all inquiries will be answered, whenever possible, by the same instructor—the nutritionist assigned to that participant. This continuity in support will help build a trusting relationship. It is believed that this close connection will enhance motivation and contribute to more personalized care. All support will be provided nonjudgmentally, with respect and empathy.

7.6 Support Center

A support center will be established to promote individualized monitoring and to centralize the distribution of guidance and tasks pre-defined by the program. Assistance will be available from Monday to Friday, between 8:00 a.m. and 6:00 p.m., via the WhatsApp application, selected due to its widespread use among the Brazilian population regardless of socioeconomic status.

7.6.1 Question Channel

To offer personalized support in a manageable way for the team, participants will be encouraged to contact the support center whenever questions arise, particularly regarding their eating routines. Messages may be sent in whichever format is most comfortable for the participant—text, voice recordings, or photos of

meals, encouraging open communication and fostering an empathetic, welcoming, and continuous relationship.

7.6.2 Adherence Channel

Program adherence will be monitored through weekly check-in messages sent by the support center. Participants will be invited to self-evaluate their week using a simple 1-to-3 scale, based on three criteria: eating pattern, dietary self-monitoring, and physical activity self-monitoring. The scale is defined as follows:

- 1 = poor
- 2 = good
- 3 = excellent

Whenever a participant rates any criterion as **1**, the instructor will initiate a conversation with practical suggestions to support improvement in that area. If the rating remains at **2** for more than two consecutive weeks, participants will be encouraged to progress to a **3** through individualized goals adapted to their daily routines.

Interaction example:

> **Patient:** “Good afternoon. My eating habits are a 3, food self-monitoring is a 2, and physical activity is a 1.”
> **Instructor:** “I'm glad to hear your eating is going well and that you’ve been consistently tracking your meals. I noticed physical activity has been more challenging lately. How about we talk about what might be affecting that?”
> **Patient:** “I just don’t feel like it.”
> **Instructor:** “Would you be open to going for a walk? Having someone to join you can really help. Walking with a neighbor or family member might make the activity feel lighter and more enjoyable.”
> **Patient:** “I’ll give it a try.”
> **Next week...**
> **Patient:** “Everything’s great! I started walking with my neighbor in the late afternoon. We’ve been going every day. I feel more motivated.”

Not always will the dialogue with participants flow easily or immediately. In such cases, new strategies must be constantly developed and proposed, even if it becomes necessary to repeat the same guidance at different times. The focus will remain on supporting the participant until some positive change

becomes possible, even if far from ideal. Every small step forward will be acknowledged and valued, and the follow-up will continue empathetically, aligned with the participant’s real possibilities at that moment.

This model of interaction will be fundamental to promote weekly adjustments and strengthen the bond between participants and lifestyle coaches. When necessary, new strategies will be suggested individually to maintain adherence to the treatment.

7.7 Weekly Structure of the Program

The program will be developed in a hybrid format, lasting a total of 20 weeks. Activities will be strategically distributed among group sessions (both in-person and online), educational videos, and follow-up messages, following an alternating schedule designed to promote ongoing engagement and content reinforcement. This structure was planned to optimize participants' time, maintain weekly contact, and encourage adherence to the program. Below is the weekly interaction schedule:

Week	Format	Topic
0	In-person session	Welcome, goal setting, and distribution of printed materials
1	Follow-up message	Reflection and goal alignment
2	Video	Healthy eating
3	Follow-up message	Practical tips for healthy eating
4	Online session	Why do we gain weight and how do we lose it?
5	Follow-up message	Strategies to reduce calories
6	Video	Physical activity
7	Follow-up message	Physical activity in practice
8	Online session	Take control of your environment
9	Follow-up message	Stimulus control
10	Video	Energy balance
11	Follow-up message	Energy balance review
12	Online session	Planning and skills for eating out
13	Follow-up message	Meal organization and planning
14	Video	Socials events
15	Follow-up message	Social events and adherence to the plan
16	Online session	Stress management and problem solving
17	Follow-up message	Dealing with negative thoughts
18	Video	Overeating
19	Follow-up message	Overcoming setbacks and maintaining consistency
20	In-person session	Closing session, motivation, and long-term maintenance

7.7.1 In-person session

During the intervention, two in-person group sessions will be held: the first in week 0 (baseline) and the second in week 20 (end of the program). These sessions will serve to collect data, foster participant integration, strengthen adherence, and align the proposed strategies.

**In-Person session – Week 0: Welcome, goal setting, and distribution of printed materials**
The first PRAMEV session will be an introductory group meeting held in person, lasting approximately 60 minutes. Its main objective is to engage participants, present the structure of the intervention, and align expectations and responsibilities.<br><br>**During the session, the following topics will be addressed:**<br>• **Introduction of the team,** including the identification of the lifestyle instructors, their professional backgrounds, and each member's role in the program.<br>• **Presentation of PRAMEV's main goal,** with emphasis on the 5% body weight reduction target, increased physical activity, and the adoption of sustainable healthy behaviors.<br>• **Explanation of the intervention structure,** detailing the 20-week schedule, including the types of sessions (in-person and online), the delivery of videos and follow-up messages, and the operation of the support center.<br>• **Introduction to key behavioral components for successful outcomes,** such as self-monitoring, self-assessment, meal planning, stimulus control, and overcoming daily barriers.<br>• **Presentation of group participation rules,** such as confidentiality, punctuality, mutual respect, active engagement, and the commitment not to share program content with third parties.<br>• **Establishment of mutual commitments between the team and participants,** highlighting the active role of the patient in adhering to the program and the continuous support to be provided by the lifestyle instructors.<br>• **Distribution of printed materials,** including the tracking booklet for recording food intake, physical activity, and body weight.<br>• **Instructions for the first assignment (homework),** which will consist of beginning daily records of meals, physical activity, and body weight, following the detailed guidance presented during the session.

In-Person session – Week 20: Closure, motivation, and maintenance

The second in-person session will take place in week 20 of the intervention, lasting approximately 60 minutes. Its main objective is to acknowledge individual progress, reinforce motivation for maintaining the acquired habits, and guide participants on continuing lifestyle changes after the program ends.

During the session, the following topics will be addressed:

- **Recognition of individual achievements,** with encouragement to reflect on the perceived benefits of weight loss and regular physical activity.
- **Development of new goals,** encouraging participants to set realistic and sustainable objectives for the future.
- **Review of maintenance strategies,** emphasizing the importance of variety in diet and physical activity, environmental adjustments, and the strengthening of social connections to support ongoing progress.
- **Discussion on motivation and relapses,** highlighting that maintaining results will require planning, continuous self-reflection, and strategies for managing difficulties without compromising achievements.
- **Program closure,** with expressions of gratitude from the team, reinforcement of the importance of individual effort, encouragement to continue the journey of change, and the distribution of a branded water bottle as a token of the program.

All in-person sessions will be conducted in a welcoming manner, providing space for active listening, sharing of experiences, and clarification of doubts.

7.7.2 Online Sessions

During the intervention, online group sessions will be conducted by nutritionists and an endocrinologist through the Google Meet platform. These sessions will be distributed throughout the 20 weeks and will alternate with the delivery of videos and follow-up messages, forming the hybrid structure of the program.

With an average duration of 45 to 60 minutes, the sessions will address core topics related to nutrition, physical activity, stimulus control, meal planning, metabolic health, and behavioral strategies for managing obesity. In addition to providing practical guidance, the online sessions will foster participant interaction, clarify doubts, and strengthen the bond with the team, contributing to adherence and continuity in the change process.

**Online session – Week 4: Why we gain weight and how we lose it?**
The first online group session will be held in the fourth week of the program and led by a nutritionist. The main goal will be to promote understanding of the relationship between caloric intake and expenditure, the energy density of foods, and the impact of these choices on body composition and weight loss.<br><br>**During the session, the following topics will be addressed:**<br><br>• **Concept of energy balance:** an explanation will be provided on how the balance between calories consumed and burned determines weight gain or loss.<br><br>• **Energy density of foods:** a distinction will be made between foods with high and low energy density, reinforcing how cooking methods can significantly affect the calorie content of meals.<br><br>• **Smart food swaps:** simple and effective substitutions will be presented to reduce the calorie density of everyday meals, such as choosing fruit instead of high-calorie desserts, reducing the use of oil in cooking, and opting for lighter versions of traditional dishes.<br><br>• **Practical and comparative examples:** a visual analysis of complete meals with different caloric compositions will be carried out to demonstrate how small changes can lead to significant differences in daily energy intake.<br><br>• **Importance of self-monitoring:** participants will be encouraged to continue recording their food intake and body weight as tools for awareness and control.<br><br>**Key message of the session:** Losing weight doesn't mean going hungry, it means making smarter and more consistent food choices to maintain a sustainable caloric deficit.

Online session – Week 8: Take control of your surroundings

The second PRAMEV online session will address the influence of the environment on food choices and physical activity levels. The class will be led by a nutritionist and will focus on raising awareness about external stimuli that influence daily behaviors, offering practical strategies to modify those stimuli to support healthier habits.

During the session, the following topics will be addressed:

- **Understanding environmental triggers**: identification of external factors that influence eating decisions and activity levels, such as the way food is arranged at home, the work environment, and spaces available for movement.
- **Home food environment**: strategies for organizing the household to support healthier choices (e.g., keeping fruit visible, avoiding stocking ultraprocessed foods, planning and preparing meals in advance).
- **Social and family environment**: how to handle social, familial, and cultural situations that may sabotage lifestyle changes, including assertive communication techniques to request support or set boundaries.
- **External environments:** how to prepare for environments such as grocery stores, restaurants, and social events, with a focus on planning and conscious decision-making.
- **Digital environment**: reflection on the impact of constant exposure to ads, social media, and food delivery platforms, and how to filter information or create a more favorable digital environment.
- **Individual action plan**: each participant will be invited to identify key environmental obstacles they face and propose at least one practical change to implement the following week.

Key message of the session: The environment we live in directly influences our choices. By recognizing and adjusting the stimuli around us, we can make it easier to adopt and maintain healthier habits.

Online session – Week 12: Planning and skills for eating out

In this online session, led by a team nutritionist, the focus will be on presenting practical strategies to maintain healthy habits in out-of-routine situations, especially during meals eaten at restaurants.

During the session, the following topics will be addressed:

• **Importance of meal planning**: participants will be guided to plan meals outside the home in advance, emphasizing the choice of restaurants that offer homemade and simple dishes, as well as searching for menu information beforehand.

• **Three key strategies for eating out:**

1. **Planning**: choose places that serve vegetables and balanced options; avoid making impulsive decisions upon arriving at the restaurant.
2. **Control**: avoid environments with "trigger foods" (such as fried items, desserts, or all-you-can-eat buffets); maintain mindful eating even during social moments.
3. **Healthy choices**: prioritize foods with lower fat content, avoid fried items, and build a healthy plate focused on vegetables, lean proteins, and balanced portions.

• **Additional tips**: participants will receive practical advice, such as observing the buffet before serving themselves, adjusting other meals during the day in case of special events, and adapting food choices without missing out on the social experience.

Key message of the session: It is possible to maintain a commitment to lifestyle change even in external environments, as long as there is planning, awareness, and effective coping strategies.

687

Online session – Week 16: Stress management and problem-solving
This session will be led by an endocrinologist from the team. The focus will be to address the impact of stress on treatment adherence, teach strategies to cope with everyday challenges, and introduce practical tools for problem-solving and managing negative thoughts.<br><br>**During the session, the following topics will be addressed:**<br><br>• **Identifying stressors**: participants will be invited to reflect on common stressful situations, such as interpersonal conflicts, work-related challenges, financial difficulties, and the lifestyle changes required by obesity treatment.<br><br>• **Stress coping strategies**: self-care practices with potential to relieve daily tension will be presented, such as listening to music, meditating, doing crafts, or engaging in enjoyable hobbies. Participants will be encouraged to consciously choose the strategies that work best for them.<br><br>• **Five-step problem-solving model**: participants will learn a practical method to approach and manage challenges: (1) list the problems, (2) brainstorm possible solutions, (3) choose the best option, (4) plan the execution, and (5) follow through. Practical examples will be used to enhance understanding and encourage application in daily life.<br><br>• **Negative and positive thoughts**: the session will discuss how thoughts influence behavior and treatment adherence. Examples of dysfunctional thinking (such as guilt or harsh self-criticism) and cognitive restructuring strategies will be presented, encouraging the replacement of these thoughts with more realistic and self-compassionate ones.<br><br>• **The cycle of negative thoughts**: participants will be guided to identify recurring patterns and reflect on how to break this cycle using positive affirmations and a shift in perspective.<br><br>**Key message of the session**: Recognizing your own limits and difficulties, learning to deal with them kindly, and seeking practical strategies to maintain emotional balance are essential aspects for sustaining lifestyle changes.

Just like the in-person sessions, the online sessions will be conducted in a welcoming and interactive manner, providing space for active listening, experience sharing, and clarification of doubts. This approach will help strengthen the bond with the team, enhance participant engagement, and support ongoing motivation throughout the lifestyle change process.

7.7.3 Video Content

As part of the continuing education strategy and reinforcement of PRAMEV's key messages, five videos will be sent throughout the intervention. These videos, each approximately one minute long, will be delivered via WhatsApp on alternating weeks.

Each video will aim to present practical guidance on nutrition, physical activity, and behavior in an accessible and straightforward way, aligned with the participants' daily lives. The decision to use short videos is intended to encourage engagement, reduce content access barriers, and maintain consistent communication with participants over the 20-week program.

**Video – Week 2: Healthy eating**
The first video will be sent to participants in the second week of the program. Its goal will be to introduce key concepts about healthy eating, highlighting practical strategies for balanced meal composition and the importance of mindful eating.<br><br>**The main topics covered in the first video will include:**<br><br>• **Healthy plate composition**: Participants will be guided on how to balance food groups on their plates, with an emphasis on vegetables, whole grains, and lean animal protein sources.<br><br>• **Foods that promote satiety**: The importance of dietary fiber and unprocessed or minimally processed foods will be highlighted as allies in the weight loss process.<br><br>• **Mindful eating**: The video will encourage participants to practice mindfulness during meals, avoiding distractions and eating slowly to improve satiety awareness and reduce overeating.<br><br>• **Reflection on eating habits**: Participants will be encouraged to self-assess behaviors that support or hinder adherence to healthy eating, with a focus on consistent—not perfect—choices over time.<br><br>The video will be accompanied by printed material (provided during the first in-person session).<br><br>**Main message**: Eating healthily and with pleasure is possible with attention, planning, and consistent choices over time.

Video – Week 6: Physical activity

In week six, the video will aim to reinforce the importance of regular physical activity and provide practical guidance on how to incorporate it into daily routines in an accessible and effective way.

The main topics covered in the second video will include:

• **The role of physical activity in health and weight loss**: The video will emphasize the benefits of regular practice, including improved energy levels, weight control, and stress reduction.

• **Components of aerobic exercise**: An explanation of the four basic principles of effective exercise will be presented: frequency, intensity, time, and type of activity.

• **Accessible and adaptable exercises**: Participants will be encouraged to engage in activities such as walking, using stairs, and incorporating movement into daily routines, respecting their individual fitness levels and available environment.

• **Weekly program goal**: The recommendation of at least 150 minutes of moderate to vigorous physical activity per week, as part of PRAMEV's goals, will be reinforced.

The video will be accompanied by printed material (provided during the first in-person session).

Main message: Regular movement is possible even with a busy schedule; the most important step is to start at your own pace and aim for consistency.

**Video – Week 10: Energy balance**
In the tenth week, the content will focus on teaching the fundamentals of energy balance and its direct relationship with weight loss and control.<br><br>**The main topics covered in the third video will include:**<br><br>• **Concept of energy balance**: A clear and simple explanation of the relationship between caloric intake and energy expenditure, highlighting how imbalances between the two can lead to weight gain or loss.<br><br>• **Importance of caloric deficit**: It will be emphasized that weight loss only occurs when the body consumes less energy than it expends, a deficit achieved through diet combined with regular physical activity.<br><br>• **Combining strategies**: Participants will be encouraged to pair a balanced diet with increased movement as an effective, sustainable, and safe way to promote weight loss and overall health improvement.<br><br>The video will be accompanied by printed material (provided during the first in-person session).<br><br>**Main message**: Understanding and applying the concept of energy balance is essential for achieving and maintaining weight loss results.

Video – Week 14: Social events

In the fourteenth week, the video will aim to prepare participants to maintain healthy habits in social and celebratory contexts by acknowledging the challenges and offering practical strategies for navigating such situations.

The main topics covered in the fourth video will include:

• **Acknowledging difficulties**: Validation of emotions and situations related to social events, such as birthdays, gatherings, and holidays, where there is often an abundance of high-calorie foods and social pressure to eat.

• **Importance of planning**: Participants will be encouraged to prepare in advance by avoiding arriving too hungry, mentally reviewing their personal goals, and deciding ahead of time how to deal with temptations.

• **Coping strategies**: Practical suggestions will be presented, such as assembling a balanced plate early on, practicing moderation rather than restriction, and focusing on social interaction as the main purpose of the event.

• **Awareness of emotional eating and guilt**: Mindful eating will be encouraged during events, along with self-compassion in case of off-plan choices, reinforcing the idea that consistency matters more than perfection.

The video will be accompanied by printed material (provided during the first in-person session).

Main message: It's possible to enjoy social moments with balance and pleasure without compromising health goals, planning and awareness are key.

Video – Week 18: Overeating

In the eighteenth week, the final educational video will offer support and strategies for dealing with dietary slip-ups, emphasizing self-care and long-term consistency.

The main topics covered in the fifth video will include:

• **Normalizing setbacks**: The video will communicate that occasional overeating is a normal part of lifestyle change and should not lead to guilt or abandoning treatment.

• **Importance of consistency**: Sustainable results come from habit consistency, not perfection. One isolated episode does not undo prior progress.

• **Reflection on triggers**: Participants will be encouraged to identify situations or emotions, such as stress, fatigue, food monotony, or social events, that may lead to overeating.

• **Recovery strategies**: Guidance will be given on how to pause, reorganize, and resume planning without self-punishment. Suggestions will include varying recipes, seeking support from the team, and reviewing goals.

Main message: Slip-ups are part of the journey, what matters most is getting back on track with kindness, balance, and consistency.

The videos will serve as a complement to the topics addressed in group sessions, online meetings, and follow-up messages, providing distinct content that broadens the program's range of strategies.

7.7.4 Follow-up messages

As part of PRAMEV's continuous follow-up strategy, participants will receive follow-up messages during odd-numbered weeks, sent via WhatsApp. These messages will aim to reinforce the content addressed in the previous week, promoting reflection, motivation, and engagement with the proposed goals.

It is important to note that these follow-up messages are distinct from the weekly verification messages sent through the adherence channel (see section 7.4.2), in which participants will perform a standardized self-assessment based on three criteria: eating pattern, dietary intake self-monitoring, and physical activity self-monitoring.

The following is a description of the follow-up messages sent during the odd-numbered weeks of the program.

Follow-up message – Week 1: Reflection and goal alignment

In the first follow-up week, participants will be guided to reflect on their personal goals and motivations regarding the program. The message sent will aim to strengthen initial engagement and help participants build a clear sense of purpose for their lifestyle change journey.

Participants will be encouraged to respond to the following prompts:

1. Why is this program important to me?
2. What do I hope to achieve by participating in this program?
3. How can eating healthier and being more active help me?

These reflections are designed to stimulate individual awareness, foster commitment to the process, and strengthen the connection between the participant and the PRAMEV initiative.

Follow-up message – Week 3: Practical guidelines for healthy eating

In the third week of the intervention, participants will be encouraged to review the printed material on healthy eating, with a focus on the food substitution list, portion sizes, and caloric content of foods. The aim is to reinforce understanding of the concepts previously introduced and to stimulate the practical application of the guidelines in daily life.

The team will also emphasize that it’s normal to face challenges at the beginning of a lifestyle change, offering encouragement and emotional support to help participants cope with any difficulties. The goal is to consolidate the adaptation process to new eating, exercise, and self-monitoring routines.

Follow-up message – Week 5: Strategies to reduce calories

The follow-up message for week five will reinforce the importance of balanced eating. Participants will be encouraged to identify high-calorie foods, those rich in sugar and fat, present in their daily routines. As a practical task, they will be guided to apply the content from the previous session by replacing these high-calorie items with lower-calorie options, as taught in session 4.

Follow-up message – Week 7: Physical activity in practice

In the seventh week, the focus will be on encouraging regular physical activity. Participants who have not yet started will be encouraged and guided to begin with small amounts, with gradual progression. For those already engaging in physical activity, the challenge will be to maintain consistency and, if possible, increase either the intensity or frequency. The message will also reinforce the importance of respecting individual health conditions and always following medical guidance. The team will remain available to help participants adapt physical activity to their routines.

Follow-up message – Week 9: Stimulus control

The follow-up message for week nine will revisit the topic of food cues, highlighting how external stimuli influence eating behavior. Participants will be encouraged to avoid keeping high-calorie foods at home or in environments that promote impulsive eating. The practical task will be to apply what was learned by replacing a negative stimulus with a positive one, as a way to strengthen self-control when facing eating triggers.

**Follow-up message – Week 11: Review on energy balance**
The follow-up message for week eleven will reinforce the importance of reviewing the printed material on energy balance, previously provided. The team will emphasize that putting the recommendations into practice is essential for consolidating new habits. The message will also offer support for clarifying doubts, reinforcing the availability of individualized guidance throughout the intervention.

**Follow-up message – Week 13: Meal planning and organization**
The follow-up message for week thirteen will aim to encourage participants to plan their meals as a key strategy for changing eating behavior. The message will highlight the benefits of organization in optimizing time, improving dietary quality, and facilitating the achievement of health and weight goals. Participants will be reminded that, while it may require initial effort, planning will lead to time savings and greater control over food choices.

**Follow-up message – Week 15: Social events and program adherence**
The follow-up message for week fifteen will encourage participants to review the content from the session on how to handle social events. The goal will be to reinforce that maintaining the practices learned, even in social situations, is a key part of changing eating behavior. The message will emphasize that applying the strategies discussed during the session will strengthen autonomy and self-control in environments that traditionally involve more challenging food choices.

**Follow-up message – Week 17: Dealing with negative thoughts**
In week seventeen, participants will be invited to reflect on how thoughts influence behavior. The message will address the difference between positive and negative thoughts and will encourage a more kind and realistic attitude toward oneself when facing setbacks. The team will reinforce the importance of replacing self-critical thoughts with positive affirmations, fostering an internal environment that supports change and persistence.

Follow-up message – Week 19: Overcoming challenges and staying consistent
In the nineteenth and final week of follow-up messages, participants will be encouraged to maintain the healthy habits acquired throughout the intervention. They will be invited to review all the materials received in order to reinforce areas of improvement and consolidate what they have learned. The message will emphasize that the end of the program actually marks the beginning of a new cycle based on conscious and sustainable health choices.

7.8 Educational Materials

To reinforce the content covered in the sessions and promote participant autonomy, PRAMEV will develop a set of printed educational materials. These materials will be distributed during the first in-person group session and will accompany participants throughout the 20-week intervention.

The content will be based on the guidelines of the DPP and adapted to the Brazilian context, incorporating recommendations from the Dietary Guidelines for the Brazilian Population and the Brazilian Food Pyramid. The materials will include guidance on healthy eating, physical activity, behavioral strategies, and planning.

To support self-monitoring, each participant will receive a journal to record their daily food intake, physical activity, and body weight.

All materials will be attached at the end of this protocol, organized according to the week they are used during the program.

7.9 Compensation for Participation

Participants will not receive any financial compensation for taking part in the study.

However, they will be allowed to keep the materials provided as part of the intervention, including:

- Printed educational materials related to the program;
- Food and physical activity tracking journals;
- A water bottle with the program’s logo.

8 DATA ANALYSIS

8.1 Statistical Analysis Strategies

The normality of continuous variables will be assessed using the Shapiro–Wilk test. Baseline characteristics of the sample will be described as mean and standard deviation for normally distributed variables, or as median and interquartile range for variables with skewed distribution. Categorical variables will be presented as absolute and relative frequencies (%).

Group comparisons at baseline will be performed using Student's t-test or the Mann–Whitney U test, depending on data distribution, for continuous variables, and the chi-square test (χ^2) for categorical variables.

The primary analysis will follow the intention-to-treat principle. Missing data at week 20 will be handled using multiple imputation by chained equations, under the assumption of missing at random. The choice of this method is justified by its robustness and wide recommendation in clinical trials, as it preserves data variability and reduces imputation bias. The plausibility of the missing at random assumption will be assessed based on the pattern and proportion of missing data.

Changes over time (Δ = week 20 − week 0) will be analyzed based on data distribution, using Student's t-test for normally distributed variables or the Mann–Whitney U test when appropriate.

A per-protocol analysis will also be performed, including only participants who completed the 20-week intervention in full.

Longitudinal changes will be evaluated using linear mixed-effects models, with a random intercept for each participant and fixed effects for time, group, and the time × group interaction. These models will account for repeated measures and within-subject correlation. Primary and secondary outcomes (e.g., weight, body mass index, waist circumference, body fat percentage) will be modeled assuming normally distributed residuals and an independent covariance structure. When appropriate, models will be adjusted for potential confounding covariates, such as age, sex, baseline BMI, and education level.

To explore the association between adherence and intervention effectiveness, a dose–response analysis will be conducted. Adherence will be defined as the percentage of sessions completed (both in-person and online), and its association with changes in clinical and psychosocial outcomes (e.g., weight, BMI, DASS-21 scores, ECAP) will be assessed using Spearman's correlation, with 95% confidence intervals estimated by bootstrap resampling (1,000 iterations).

Response analyses will be conducted using logistic regression to estimate the probability of achieving ≥5% weight loss, adjusted for baseline covariates.

Exploratory within-group analyses (week 0 vs. week 20) will be performed using paired t-tests (for normally distributed variables) or Wilcoxon signed-rank tests (for nonparametric variables), including only participants with complete paired data.

Additionally, subgroup analyses may be conducted based on age range or generational cohort (e.g., Baby Boomers, Generation X, Y, Z). Comparisons between subgroups will be performed using the chi-square test (χ^2).

In the case of multiple secondary comparisons, correction methods such as Bonferroni or false discovery rate will be applied to control the risk of Type I error.

All tests will be two-tailed, with a statistical significance level set at $p < 0.05$.

9 QUALITY MONITORING OF THE INTERVENTION

As this is a single-center study, the program coordinator will be directly involved in the following processes:

- Participant screening;
- Obtaining informed consent;
- Data collection.

The coordinator's presence is intended to ensure adherence to the study protocol and the quality of operational procedures, providing continuous support to the research team and addressing any questions that may arise during study implementation.

10 DATA SECURITY

Paper-based data will be stored in locked cabinets located in a restricted-access room at the research unit of the Obesity Outpatient Clinic, Division of Endocrinology and Metabolism, at the Clinical Hospital of the University of São Paulo Medical School (HCFMUSP). Electronic data will be stored on secure institutional servers with restricted access granted only to authorized researchers, and on encrypted backup media managed by the research team.

All extracted data will be used exclusively for scientific purposes and will undergo anonymization procedures, including the removal of personal identifiers and replacement with internal codes. These measures are intended to ensure participant privacy and minimize the risk of reidentification, in accordance with Resolution No. 466/2012 of the Brazilian National Health Council (item III.1.h) and the Brazilian General Data Protection Law – LGPD (Law No. 13,709/2018, Article 7, IV and Article 11, II).

Data will not be shared with third parties, except when required by legal or regulatory authorities. After study completion, all data will be stored for the period mandated by Brazilian ethical and regulatory standards, under the responsibility of the research team, and will subsequently be securely destroyed.

Although the intervention is considered low-risk, the study will be monitored through periodic reviews by the Research Ethics Committee of the University of São Paulo Medical School, in compliance with Resolution CNS No. 466/2012. Any adverse events, incidents, or protocol deviations will be documented and reported to the committee for appropriate evaluation and guidance.

11 REFERENCES

1. Luppino FS, de Wit LM, Bouvy PF, Stijnen T, Cuijpers P, Penninx BW, et al. Overweight, obesity, and depression: a systematic review and meta-analysis of longitudinal studies. Arch Gen Psychiatry. 2010;67(3):220-9.

2. Haslam DW, James WP. Obesity. Lancet. 2005;366(9492):1197-209.

3. Ng M, Fleming T, Robinson M, Thomson B, Graetz N, Margono C, et al. Global, regional, and national prevalence of overweight and obesity in children and adults during 1980-2013: a systematic analysis for the Global Burden of Disease Study 2013. Lancet. 2014;384(9945):766-81.

4. World Health Organization (WHO) 2016 [Available from: https://www.who.int/news-room/fact-sheets/detail/obesity-and-overweight .

5. IBGE IBdGeE-. Atenção primária à saúde e informações antropométricas 2019.

6. Raaijmakers LC, Pouwels S, Thomassen SE, Nienhuijs SW. Quality of life and bariatric surgery: a systematic review of short- and long-term results and comparison with community norms. Eur J Clin Nutr. 2017;71(4):441-9.

7. Gloy VL, Briel M, Bhatt DL, Kashyap SR, Schauer PR, Mingrone G, et al. Bariatric surgery versus non-surgical treatment for obesity: a systematic review and meta-analysis of randomised controlled trials. Bmj. 2013;347:f5934.

8. Nilson EAF, Andrade R, de Brito DA, de Oliveira ML. Costs attributable to obesity, hypertension, and diabetes in the Unified Health System, Brazil, 2018. Rev Panam Salud Publica. 2020;44:e32.

9. Wadden TA, West DS, Delahanty L, Jakicic J, Rejeski J, Williamson D, et al. The Look AHEAD study: a description of the lifestyle intervention and the evidence supporting it. Obesity (Silver Spring). 2006;14(5):737-52.

10. Eight-year weight losses with an intensive lifestyle intervention: the look AHEAD study. Obesity (Silver Spring). 2014;22(1):5-13.

11. Curry SJ, Krist AH, Owens DK, Barry MJ, Caughey AB, Davidson KW, et al. Behavioral Weight Loss Interventions to Prevent Obesity-Related Morbidity and Mortality in Adults: US Preventive Services Task Force Recommendation Statement. Jama. 2018;320(11):1163-71.

12. Yanovski SZ, Yanovski JA. Long-term drug treatment for obesity: a systematic and clinical review. Jama. 2014;311(1):74-86.

13. Wadden TA, Butryn ML, Byrne KJ. Efficacy of Lifestyle Modification for Long-Term Weight Control. Obesity Research. 2004;12(S12):151S-62S.

14. Divino V, Ramasamy A, Anupindi VR, Eriksen KT, Olsen AH, DeKoven M, et al. Complication-specific direct medical costs by body mass index for 13 obesity-related complications: a retrospective database study. J Manag Care Spec Pharm. 2021;27(2):210-22.

15. Armenta-Guirado B, Martínez-Contreras T, Candia-Plata MC, Esparza-Romero J, Martínez-Mir R, Haby MM, et al. Effectiveness of the Diabetes Prevention Program for Obesity Treatment in Real World Clinical Practice in a Middle-Income Country in Latin America. Nutrients. 2019;11(10).

16. The Diabetes Prevention Program. Design and methods for a clinical trial in the prevention of type 2 diabetes. Diabetes Care. 1999;22(4):623-34.

17. The Diabetes Prevention Program (DPP): description of lifestyle intervention. Diabetes Care. 2002;25(12):2165-71.

18. Knowler WC, Barrett-Connor E, Fowler SE, Hamman RF, Lachin JM, Walker EA, et al. Reduction in the incidence of type 2 diabetes with lifestyle intervention or metformin. N Engl J Med. 2002;346(6):393-403.

19. Salas-Salvadó J, Díaz-López A, Ruiz-Canela M, Basora J, Fitó M, Corella D, et al. Effect of a Lifestyle Intervention Program With Energy-Restricted Mediterranean Diet and Exercise on Weight Loss and Cardiovascular Risk Factors: One-Year Results of the PREDIMED-Plus Trial. Diabetes Care. 2019;42(5):777-88.

20. Porca C, Rodriguez-Carnero G, Tejera C, Andujar P, Casanueva FF, Crujeiras AB, et al. Effectiveness to promote weight loss maintenance and healthy lifestyle habits of a group educational intervention program in adults with obesity: IGOBE program. Obes Res Clin Pract. 2021;15(6):570-8.

21. Wadden TA, Neiberg RH, Wing RR, Clark JM, Delahanty LM, Hill JO, et al. Four-year weight losses in the Look AHEAD study: factors associated with long-term success. Obesity (Silver Spring). 2011;19(10):1987-98.

22. Estatística IBdGe. PNAD Contínua TIC 2019: internet chega a 82,7% dos domicílios do país: Agência de Notícias IBGE; 2020 [Available from: https://agenciadenoticias.ibge.gov.br/agencia-sala-de-imprensa/2013-agencia-de-noticias/releases/30521-pnad-continua-tic-2019-internet-chega-a-82-7-dos-domicilios-do-pais.

23. AR F. *Anthropometric standards for the assessment of growth and nutritional*

status. Ann Arbor: The Universitty of Michigan Press1999.

24. World Health Organization

(WHO).Obesity: preventing and managing the global epidemic 2000. 252 p.

25. Whelton PK, Carey RM, Aronow WS, Casey DE, Collins KJ, Dennison Himmelfarb C, et al. 2017 ACC/AHA/AAPA/ABC/ACPM/AGS/APhA/ASH/ASPC/NMA/PCNA Guideline for the Prevention, Detection, Evaluation, and Management of High Blood Pressure in Adults: Executive Summary. A Report of the American College of Cardiology/American Heart Association Task Force on Clinical Practice Guidelines. 2018;71(19):2199-269.

26. Pate RR, O'Neill JR, Lobelo F. The evolving definition of "sedentary". Exerc Sport Sci Rev. 2008;36(4):173-8.

27. Piercy KL, Troiano RP, Ballard RM, Carlson SA, Fulton JE, Galuska DA, et al. The Physical Activity Guidelines for Americans. Jama. 2018;320(19):2020-8.

28. Ainsworth BE, Haskell WL, Herrmann SD, Meckes N, Bassett DR, Jr., Tudor-Locke C, et al. 2011 Compendium of Physical Activities: a second update of codes and MET values. Med Sci Sports Exerc. 2011;43(8):1575-81.

29. Fisberg RM, Marchioni DML, Colucci ACA. Avaliação do consumo alimentar e da ingestão de nutrientes na prática clínica. Arquivos Brasileiros de Endocrinologia & Metabologia. 2009;53:617-24.

30. Monteiro CA, Cannon G, Moubarac JC, Levy RB, Louzada MLC, Jaime PC. The UN Decade of Nutrition, the NOVA food classification and the trouble with ultra-processing. Public Health Nutr. 2018;21(1):5-17.

31. Freitas S, Lopes CS, Coutinho W, Appolinario JC. Tradução e adaptação para o português da Escala de Compulsão Alimentar Periódica. Brazilian Journal of Psychiatry. 2001;23:215-20.

32. Gormally J, Black S, Daston S, Rardin D. The assessment of binge eating severity among obese persons. Addict Behav. 1982;7(1):47-55.

33. Vignola RC, Tucci AM. Adaptation and validation of the depression, anxiety and stress scale (DASS) to Brazilian Portuguese. J Affect Disord. 2014;155:104-9.

34. Jensen MD, Ryan DH, Apovian CM, Ard JD, Comuzzie AG, Donato KA, et al. 2013 AHA/ACC/TOS guideline for the management of overweight and obesity in adults: a report of the American College of Cardiology/American Heart Association Task Force on Practice Guidelines and The Obesity Society. Circulation. 2014;129(25 Suppl 2):S102-38.

35. Stevens VJ, Obarzanek E, Cook NR, Lee IM, Appel LJ, Smith West D, et al. Long-term weight loss and changes in blood pressure: results of the Trials of Hypertension Prevention, phase II. Ann Intern Med. 2001;134(1):1-11.

36. Williamson DA, Bray GA, Ryan DH. Is 5% weight loss a satisfactory criterion to define clinically significant weight loss? Obesity (Silver Spring). 2015;23(12):2319-20.

37. Wing RR, Lang W, Wadden TA, Safford M, Knowler WC, Bertoni AG, et al. Benefits of modest weight loss in improving cardiovascular risk factors in overweight and obese individuals with type 2 diabetes. Diabetes Care. 2011;34(7):1481-6.

38. Unick JL, Beavers D, Jakicic JM, Kitabchi AE, Knowler WC, Wadden TA, et al. Effectiveness of lifestyle interventions for individuals with severe obesity and type 2 diabetes: results from the Look AHEAD trial. Diabetes Care. 2011;34(10):2152-7.

39. Bull FC, Al-Ansari SS, Biddle S, Borodulin K, Buman MP, Cardon G, et al. World Health Organization 2020 guidelines on physical activity and sedentary behaviour. Br J Sports Med. 2020;54(24):1451-62.
