# Supplementary material for: A Hybrid Lifestyle Program and Metabolic Health in Adults With Obesity: The PRAMEV Randomized Clinical Trial
Source: JAMA Netw Open. 2026 Aug 11;9(8):e2626884. doi: 10.1001/jamanetworkopen.2026.26884 (PMC13463168; doi:10.1001/jamanetworkopen.2026.26884)
Supplement: Supplementary file 2 [file jamanetwopen-e2626884-s002.pdf]

Supplemental Online Content

Souza de Menezes Amorim Coelho G, Fernandes AE, Pepe RB, et al. A hybrid lifestyle program and metabolic health in adults with obesity: the PRAMEV randomized clinical trial. *JAMA Netw Open*. 2026;9(8):e2626884. doi:10.1001/jamanetworkopen.2026.26884

eTable 1. Sensitivity Analyses for Anthropometric Outcomes Under MNAR Assumptions (ITT Analysis)

eTable 2. Changes in Anthropometric, Clinical, and Laboratory Variables in the Per-Protocol Analysis

eTable 3. Clinical, Behavioral, and Dietary Measures at Baseline and Week 20 by Treatment Group

eTable 4. Correlation Between Session Attendance and Changes in Clinical and Psychosocial Outcomes

eTable 5. Baseline Characteristics According to Dropout Status by Study Group

eFigure 1. Anti-Obesity Medication Use by Group and Over Time

eFigure 2. Study Dropout Rates According to Reason and Participant Generation Protocol of Usual Medical Care

This supplemental material has been provided by the authors to give readers additional information about their work.

© 2026 Souza de Menezes Amorim Coelho G et al. *JAMA Network Open*.

eTable 1. Sensitivity Analyses for Anthropometric Outcomes Under MNAR Assumptions (ITT Analysis)

Outcome	MNAR Scenario	OR or AMD (95% CI) [a]	P value
**≥5% weight loss [b]**			
MAR (reference)	0 kg	1.34 (0.61-2.92)	.46
MNAR penalty	+0.5 kg	1.32 (0.61-2.89)	.47
	+1.0 kg	1.20 (0.54-2.66)	.64
	+2.0 kg	1.07 (0.48-2.41)	.86
**Weight change, kg [c]**			
MAR (reference)	0 kg	0.6 (-0.8 to 2.0)	.42
MNAR penalty	+0.5 kg	0.4 (-1.0 to 1.8)	.59
	+1.0 kg	0.2 (-1.2 to 1.6)	.80
	+2.0 kg	-0.2 (-1.6 to 1.2)	.78
**Weight change, % [c]**			
MAR (reference)	0 kg	0.6 (-0.7 to 1.9)	.37
MNAR penalty	+0.5 kg	0.4 (-0.9 to 1.7)	.56
	+1.0 kg	0.2 (-1.1 to 1.5)	.79
	+2.0 kg	-0.2 (-1.5 to 1.1)	.74
**BMI change, kg/m² [c]**			
MAR (reference)	0 kg	0.5 (-0.1 to 1.2)	.11
MNAR penalty	+0.5 kg	0.4 (-0.2 to 1.1)	.20
	+1.0 kg	0.3 (-0.3 to 1.0)	.32
	+2.0 kg	0.1 (-0.5 to 0.8)	.69
**Waist circumference change, cm [c]**			
MAR (reference)	0 cm	1.6 (-0.3 to 3.4)	.10
MNAR penalty	+1 cm	0.6 (-1.3 to 2.4)	.55
	+2 cm	-0.4 (-2.3 to 1.4)	.64
	+3 cm	-1.4 (-3.3 to 0.4)	.12

Abbreviations: AMD, adjusted mean difference; BMI, body mass index; CI, confidence interval; ITT, intention-to-treat; MAR, missing at random; MNAR, missing not at random; OR, odds ratio.
[a] OR reported for ≥5% weight loss; AMD reported for all continuous outcomes. All models were adjusted for baseline values, age, and sex.

© 2026 Souza de Menezes Amorim Coelho G et al. *JAMA Network Open*.

[b] MAR scenario represents the primary ITT analysis under the missing-at-random assumption using multiple imputation by chained equations. MNAR sensitivity analyses applied incremental weight penalties (+0.5 to +2.0 kg) to imputed values in the intervention group to assess robustness of findings.
[c] MNAR sensitivity analyses for waist circumference applied incremental penalties of +1 to +3 cm; all other continuous outcomes used +0.5 to +2.0 kg penalties.

© 2026 Souza de Menezes Amorim Coelho G et al. *JAMA Network Open*.

eTable 2. Changes in Anthropometric, Clinical, and Laboratory Variables in the Per-Protocol Analysis

Outcome	PRAMEV (Δ) [a]	n	Control Group (Δ) [a]	n	P value
Weight, %	-2.2 (-4.1 to 0.7)	90	-0.7 (-2.9 to 1.3)	103	.03
Desired weight, kg	3.0 (0.0 to 6.5)	58	0.0 (0.0 to 5.0)	81	.006
BMI, kg/m²	-0.9 (-2.1 to 0.3)	90	-0.4 (-1.2 to 0.5)	103	.03
WC, cm	-4.3 (5.8)	41	-1.7 (5.3)	66	.03
SBP, mmHg	0.4 (11.4)	35	-3.1 (12.0)	64	.16
DBP, mmHg	1.9 (18.3)	36	-3.0 (18.8)	64	.20
HR, bpm	8.0 (1.5 to 15.0)	23	14.0 (1.0 to 19.0)	35	.28
TEE, kcal/day	-1.5 (-34.5 to 18.5)	56	13.0 (-7.0 to 27.0)	81	.004
SMM, kg	0.0 (-0.9 to 0.4)	56	0.3 (-0.2 to 0.7)	81	.007
BF, %	-0.8 (-2.3 to 0.3)	56	-0.7 (-2.0 to 0.2)	80	.59
Physical activity energy expenditure, kcal/day	0.0 (0.0 to 188.4)	57	0.0 (-0.1 to 118.5)	80	.26
AST, U/L	-1.0 (-3.0 to 2.5)	39	-1.0 (-3.8 to 1.8)	54	.58
ALT, U/L	-2.0 (-8.0 to 1.8)	38	-3.0 (-6.8 to 2.0)	54	.89
Glucose, mg/dL	-1.0 (-9.0 to 8.0)	45	2.0 (-7.0 to 10.0)	63	.19
HbA1c, %	0.0 (-0.1 to 0.2)	44	0.1 (-0.1 to 0.2)	66	.55
LDL-C, mg/dL	-4.5 (-17.0 to 8.5)	50	4.0 (-7.3 to 14.3)	68	.05
HDL-C, mg/dL	3.2 (7.5)	49	1.2 (7.0)	67	.14
Serum total cholesterol, mg/dL	0.5 (-17.8 to 11.5)	50	6.0 (-14.0 to 21.5)	68	.13
Triglycerides, mg/dL	-11.0 (-32.0 to 9.0)	49	3.0 (-20.0 to 23.0)	69	.03
Stress score	-2.0 (-8.0 to 6.0)	57	-2.0 (-6.0 to 4.0)	81	.83
Depression score	-2.0 (-8.0 to 2.0)	57	-2.0 (-6.0 to 4.0)	81	.79
Anxiety score	-2.0 (-8.0 to 2.0)	57	0.0 (-6.0 to 4.0)	81	.44
BES score	-3.0 (-8.0 to 1.0)	57	-1.0 (-7.0 to 3.0)	81	.28
Total energy intake, kcal	-132.3 (-249.7 to 112.5)	18	31.7 (-339.2 to 256.8)	42	.17

© 2026 Souza de Menezes Amorim Coelho G et al. *JAMA Network Open*.

eTable 2. Changes in Anthropometric, Clinical, and Laboratory Variables in the Per-Protocol Analysis (continued)

Characteristic	PRAMEV (Δ) [a]	n	Control Group (Δ) [a]	n	P value
Total protein, %TEI	-0.4 (-1.4 to 4.1)	18	-0.1 (-2.2 to 3.7)	42	.93
Total carbohydrate, %TEI	3.8 (-2.9 to 11.8)	18	1.0 (-7.6 to 5.2)	42	.29
Total lipid, %TEI	-4.1 (-8.8 to -1.2)	18	1.3 (-9.9 to 4.8)	42	.38
Saturated fat, %TEI	-3.7 (-6.3 to -0.4)	18	-0.5 (-6.8 to 5.8)	42	.23
Monounsaturated fat, %TEI	-2.5 (-4.8 to 1.0)	18	-0.6 (-3.8 to 1.6)	42	.15
Polyunsaturated fat, %TEI	0.4 (-2.6 to 1.0)	18	0.1 (-2.0 to 1.6)	42	.93
Dietary cholesterol, %TEI	-16.7 (-95.5 to 158.3)	18	5.6 (-90.5 to 121.9)	42	.78
Total fiber, g	0.4 (-6.8 to 2.2)	18	0.6 (-4.8 to 5.9)	42	.37
G1, % TEI	3.4 (-10.3 to 19.6)	18	-0.5 (-8.8 to 22.7)	42	.93
G2, % TEI	-0.3 (-2.0 to 0.4)	18	0.0 (-1.7 to 2.5)	42	.36
G3, % TEI	0.5 (-10.9 to 8.4)	18	-4.2 (-11.6 to 5.0)	42	.70
G4, % TEI	-6.0 (-15.0 to 6.7)	18	-1.7 (-13.7 to 8.0)	42	.76

Abbreviations: BMI, body mass index; WC, waist circumference; SBP, systolic blood pressure; DBP, diastolic blood pressure; HR, heart rate; TEE, total energy expenditure; SMM, skeletal muscle mass; BF, body fat; AST, aspartate aminotransferase; ALT, alanine aminotransferase; HbA1c, hemoglobin A1c; LDL-C, low-density lipoprotein cholesterol; HDL-C, high-density lipoprotein cholesterol; BES, binge eating scale; TEI, total energy intake; G1, group 1 consists of unprocessed or minimally processed foods; G2, group 2 comprises processed culinary ingredients; G3, group 3 involves processed foods; G4, group 4 includes ultra-processed foods and beverages.

[a] Values are presented as median (IQR) or mean (SD), as appropriate. Analyses were conducted according to the per-protocol principle. Outcomes were compared between groups using the Student t test or the Mann–Whitney U test, as appropriate.

Δ indicates the change from baseline (week 0) to post-intervention (week 20).

© 2026 Souza de Menezes Amorim Coelho G et al. *JAMA Network Open*.

eTable 3. Clinical, Behavioral, and Dietary Measures at Baseline and Week 20 by Treatment Group

	PRAMEV			Control Group		
**Characteristic**	**Week 0** [a]	**Week 20** [a]	**P value**[b]	**Week 0**[a]	**Week 20**[a]	**P value**[b]
Weight, %	94.5 (83.0 to 106.6)	90.9 (80.8 to 104.4)	<.001	95.9 (88.4 to 108.4)	95.0 (87.2 to 106.8)	.02
Desired weight, kg	68.0 (60.0 to 75.0)	70.0 (65.0 to 75.0)	<.001	70.0 (65.0 to 75.0)	70.0 (65.0 to 80.0)	.11
BMI, kg/m²	37.1 (33.5 to 41.1)	35.8 (32.5 to 40.0)	<.001	37.2 (33.7 to 41.1)	36.2 (33.2 to 40.5)	.004
WC, cm	112.0 (104.9 to 121.0)	106.3 (100.4 to 118.0)	<.001	113.1 (106.0 to 122.4)	111.6 (104.3 to 119.4)	.007
SBP, mmHg	129.3 (17.0)	131.2 (16.9)	.53	124.0 (112.0 to 140.0)	120.5 (111.7 to 133.3)	.26
DBP, mmHg	82.0 (76.5 to 90.0)	81.0 (75.5 to 89.5)	.90	80.0 (70.0 to 90.0)	78.0 (73.8 to 84.0)	.05
HR, bpm	76.4 (16.2)	83.4 (11.8)	.05	78.0 (71.5 to 85.5)	90.0 (78.0 to 105.0)	<.001
TEE, kcal/day	1427.8 (208.8)	1416.4 (191.3)	.05	1449.0 (1363.0 to 1561.0)	1458.0 (1374.0 to 1575.0)	.004
SMM, kg	25.4 (23.0 to 28.2)	25.1 (23.1 to 28.1)	.13	27.4 (25.2 to 30.5)	27.7 (25.4 to 31.0)	.008
BF, %	50.0 (46.1 to 52.4)	47.7 (43.6 to 51.9)	<.001	49.1 (45.7 to 51.4)	48.2 (44.4 to 51.0)	<.001
Physical activity energy expenditure, kcal/day	0.0 (0.0 to 281.1)	183.2 (0.0 to 324.5)	.034	0.0 (0.0 to 256.2)	0.0 (0.0 to 254.5)	.50
AST, U/L	19.0 (16.0 to 24.5)	19.0 (16.0 to 22.5)	.55	18.0 (17.0 to 20.8)	18.0 (15.0 to 21.0)	.15
ALT, U/L	19.0 (13.0 to 25.8)	16.5 (13.0 to 22.8)	.02	18.5 (15.0 to 24.8)	16.0 (13.0 to 21.8)	.02
Glucose, mg/dL	92.0 (87.0 to 105.0)	93.0 (85.0 to 106.0)	.37	96.0 (87.5 to 108.0)	95.0 (84.5 to 113.0)	.39
HbA1c, %	5.6 (5.2 to 6.1)	5.6 (5.3 to 6.1)	.43	5.7 (5.3 to 6.3)	5.7 (5.4 to 6.2)	.10
LDL-C, mg/dL	107.0 (87.8 to 125.2)	93.0 (76.2 to 120.5)	.11	102.5 (80.8 to 126.5)	107.5 (84.5 to 126.0)	.17
HDL-C, mg/dL	51.0 (11.7)	54.2 (12.7)	.004	48.0 (39.0 to 55.0)	51.0 (41.5 to 56.0)	.23
Serum total cholesterol, mg/dL	180.0 (36.3)	172.0 (33.2)	.11	174.0 (145.8 to 197.0)	180.5 (157.0 to 199.3)	.33
Triglycerides, mg/dL	107.0 (82.0 to 123.0)	95.0 (81.0 to 113.0)	.01	112.0 (82.0 to 142.0)	116.0 (82.0 to 154.0)	.56
Stress score	14.0 (8.0 to 24.0)	14.0 (6.0 to 22.0)	.25	18.0 (10.0 to 28.0)	16.0 (8.0 to 26.0)	.07
Depression score	10.0 (4.0 to 24.0)	8.0 (2.0 to 20.0)	.07	12.0 (6.0 to 24.0)	8.0 (4.0 to 18.0)	.03
Anxiety score	10.0 (4.0 to 18.0)	6.0 (2.0 to 16.0)	.07	12.0 (4.0 to 20.0)	10.0 (4.0 to 18.0)	.35
BES score	11.0 (5.0 to 18.0)	9.0 (3.0 to 13.0)	.01	11.0 (6.0 to 18.0)	10.0 (4.0 to 15.0)	.17

© 2026 Souza de Menezes Amorim Coelho G et al. *JAMA Network Open*.

eTable 3. Clinical, Behavioral, and Dietary Measures at Baseline and Week 20 by Treatment Group (continued)

	PRAMEV			Control Group		
**Characteristic**	**Week 0[a]**	**Week 20[a]**	**P value[b]**	**Week 0[a]**	**Week 20[a]**	**P value[b]**
Total energy intake, kcal	1332.1 (1103.6 to 1677.3)	1204.9 (1125.6 to 1404.0)	.07	1332.9 (1201.2 to 1759.1)	1363.7 (1248.0 to 1784.9)	.92
Total protein, %TEI	18.8 (6.0)	20.5 (5.3)	.32	17.2 (14.6 to 19.6)	16.7 (13.7 to 20.9)	.78
Total carbohydrate, %TEI	49.2 (9.5)	51.9 (7.2)	.28	48.5 (44.7 to 55.4)	48.6 (8.9)	.78
Total lipid, %TEI	31.9 (5.9)	28.5 (5.9)	.07	32.6 (27.4 to 39.4)	32.5 (6.6)	.74
Saturated fat, %TEI	10.6 (2.6)	9.3 (2.2)	.08	12.3 (3.3)	11.8 (9.3 to 13.4)	.40
Monounsaturated fat, %TEI	10.3 (3.0)	7.6 (6.7-9.2)	.009	9.4 (8.2 to 13.2)	9.9 (7.7 to 10.8)	.21
Polyunsaturated fat, %TEI	6.6 (3.1)	6.0 (2.1)	.42	6.6 (2.1)	6.4 (4.9 to 7.5)	.87
Dietary cholesterol, %TEI	178.5 (148.6 to 286.1)	219.6 (120.5 to 337.3)	.97	187.7 (152.2 to 308.3)	257.1 (133.2 to 372.2)	.54
Total fiber, g	15.3 (6.8)	13.7 (5.9)	.38	15.4 (10.2 to 19.9)	16.6 (6.8)	.75
G1, % TEI	58.2 (18.3)	64.3 (16.0)	.33	54.1 (16.1)	59.5 (21.0)	.11
G2, % TEI	3.8 (3.2)	2.8 (0.1 to 5.0)	.26	1.6 (0.3 to 6.3)	3.0 (0.6 to 4.9)	.60
G3, % TEI	12.1 (5.6 to 21.7)	16.2 (13.5)	.97	18.6 (10.3 to 31.8)	12.2 (5.3 to 26.5)	.18
G4, % TEI	21.1 (14.0)	16.2 (10.2)	.22	22.4 (15.0)	16.0 (6.5 to 26.2)	.20

Abbreviations: BMI, body mass index; WC, waist circumference; SBP, systolic blood pressure; DBP, diastolic blood pressure; HR, heart rate; TEE, total energy expenditure; SMM, skeletal muscle mass; BF, body fat; AST, aspartate aminotransferase; ALT, alanine aminotransferase; HbA1c, hemoglobin A1c; LDL-C, low-density lipoprotein cholesterol; HDL-C, high-density lipoprotein cholesterol; BES, binge eating scale; TEI, total energy intake; G1, group 1 consists of unprocessed or minimally processed foods; G2, group 2 comprises processed culinary ingredients; G3, group 3 involves processed foods; G4, group 4 includes ultra-processed foods and beverages.

[a] Values are presented as median (IQR) or mean (SD), as appropriate.

[b] P values reflect within-group comparisons between week, using the paired t test or Wilcoxon signed-rank test, as appropriate.

© 2026 Souza de Menezes Amorim Coelho G et al. *JAMA Network Open*.

eTable 4. Correlation Between Session Attendance and Changes in Clinical and Psychosocial Outcomes

Outcome	Spearman rho [a]	P value [b]	95% CI
Δ Weight	0.0	.89	-0.2 to 0.2
Δ BMI	-0.2	.18	-0.2 to 0.2
Δ Waist circumference	-0.2	.14	-0.5 to 0.1
Δ DASS-Anxiety	0.1	.71	-0.2 to 0.3
Δ DASS-Depression	-0.0	.82	-0.3 to 0.2
Δ DASS-Stress	0.0	.91	-0.3 to 0.3
Δ BES	-0.1	.44	-0.4 to 0.2

Abbreviations: BMI, body mass index; DASS, Depression, Anxiety and Stress Scale; BES, Binge Eating Scale; CI, confidence interval.
[a] Spearman's rank correlation coefficient.
[b] All P values are 2-sided and were calculated using Spearman's rank correlation test.
Δ indicates the change from baseline (week 0) to post-intervention (week 20).

© 2026 Souza de Menezes Amorim Coelho G et al. *JAMA Network Open*.

eTable 5. Baseline Characteristics According to Dropout Status by Study Group

	PRAMEV			Control Group		
**Characteristic**	**Dropout (n = 38)**	**Completers (n = 59)**	**P value**	**Dropout (n = 22)**	**Completers (n = 81)**	**P value**
Sex, female, n (%)	27 (71.1%)	52 (88.1%)	.07	18 (81.8%)	75 (92.6%)	.27
Age, y	44.7 (14.7)	52.5 (13.6)	.009	40.9 (12.3)	48.0 (13.0)	.02
Weight, kg	97.8 (86.7 to 111.8)	92.5 (82.8 to 103.8)	.16	97.6 (86.6 to 112.9)	95.7 (88.4 to 108.0)	.76
BMI, kg/m²	37.0 (34.1 to 39.2)	37.0 (33.6 to 41.1)	.89	38.5 (31.4 to 41.1)	37.2 (33.8 to 40.8)	.78
Waist circumference, cm	115.0 (108.1 to 121.8)	111.1 (103.8 to 120.8)	.22	109.5 (101.8 to 119.5)	114.0 (106.0 to 122.3)	.32
Program attendance [a], %	33.3 (14.3 to 73.8)	81.0 (66.7 to 95.2)	<.001			

Abbreviations: BMI, body mass index.

Values are presented as mean (SD) or median (IQR), as appropriate. Between-group comparisons were performed using the t test for normally distributed variables, the Mann–Whitney U test for nonparametric variables, and the χ^2 test for categorical variables.

[a] Program attendance was applicable only to the PRAMEV group, as the control group did not participate in a structured program.

Program attendance (%) was calculated based on participant engagement, including interactions with messages and attendance at scheduled sessions.

© 2026 Souza de Menezes Amorim Coelho G et al. *JAMA Network Open*.

eFigure 1. Anti-Obesity Medication Use by Group and Over Time

A, Between-Group Comparison at Baseline and Week 20

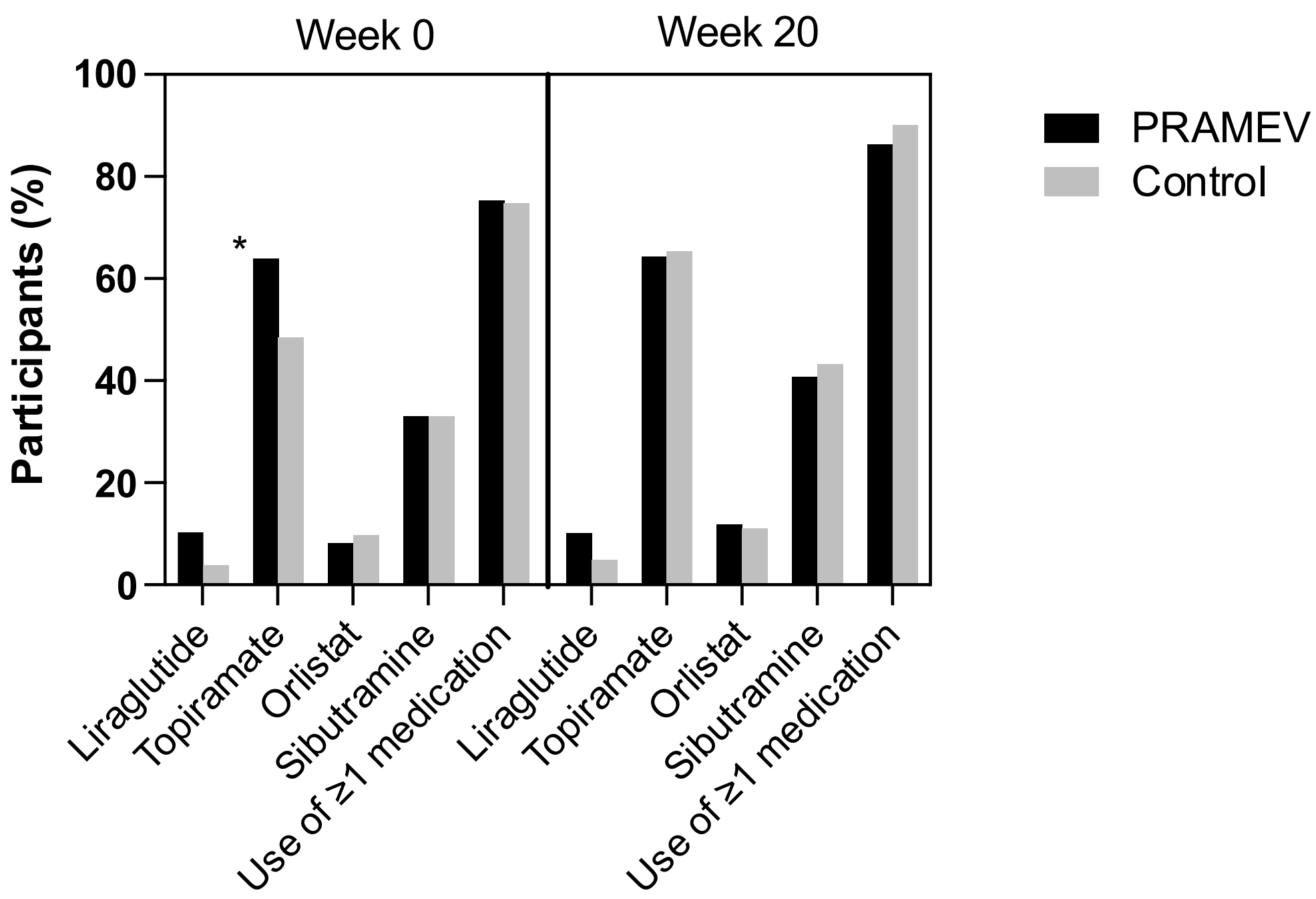

B, Within-Group Comparison from Baseline to Week 20

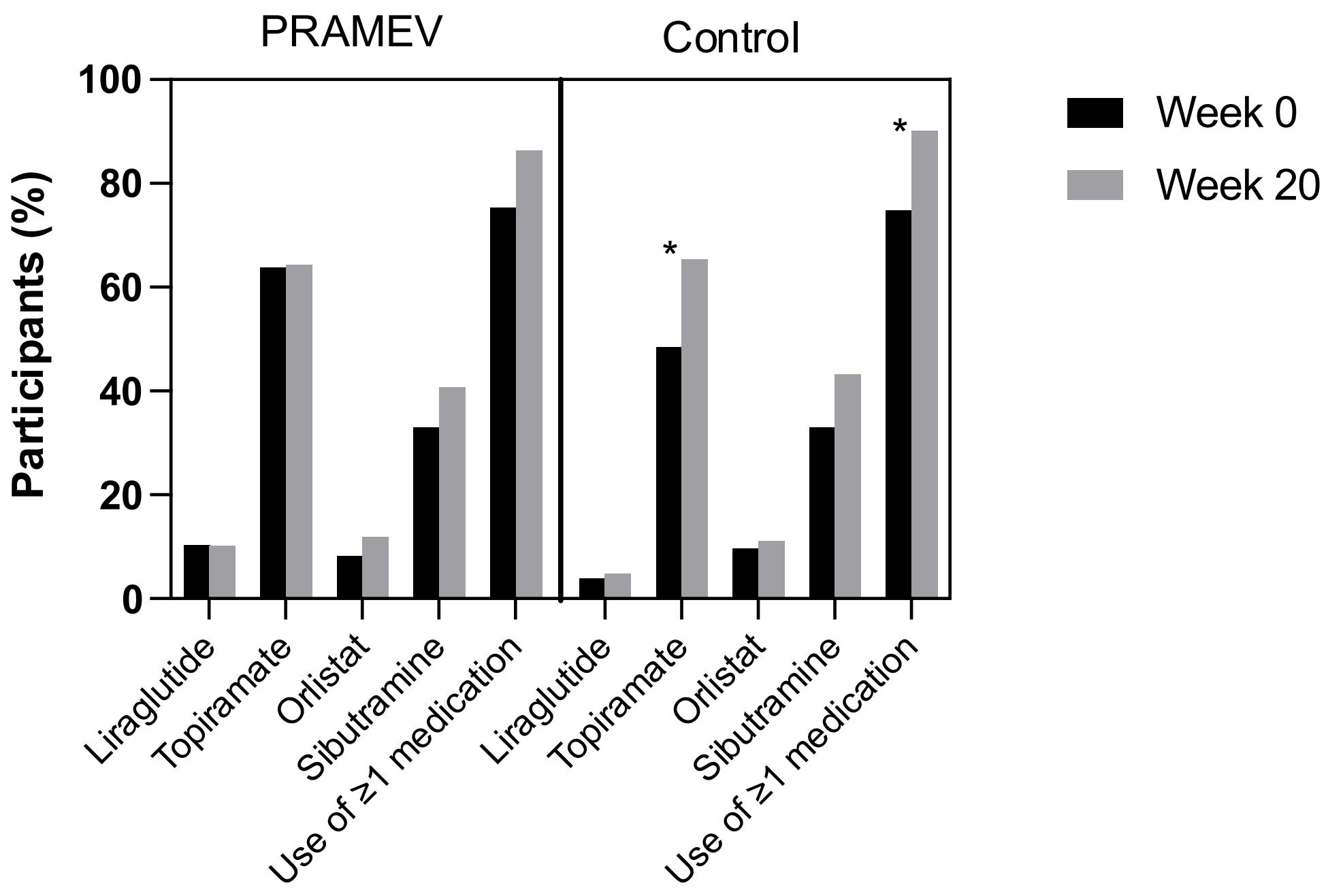

A, Percentage of participants using each medication (liraglutide, topiramate, orlistat, and sibutramine) at week 0 and week 20, comparing the PRAMEV with the control group. * Indicates statistically significant difference between groups at week 20 only (P < .05, Fisher exact test).

© 2026 Souza de Menezes Amorim Coelho G et al. *JAMA Network Open*.

B, Percentage of participants using each medication at baseline and at week 20 within each group (PRAMEV and control). * Indicates statistically significant difference between time points within the same group ($P < .05$, Fisher exact test). "≥1 medication" indicates use of at least 1 anti-obesity medication.

© 2026 Souza de Menezes Amorim Coelho G et al. *JAMA Network Open*.

eFigure 2. Study Dropout Rates According to Reason and Participant Generation

A, Reasons for Dropout

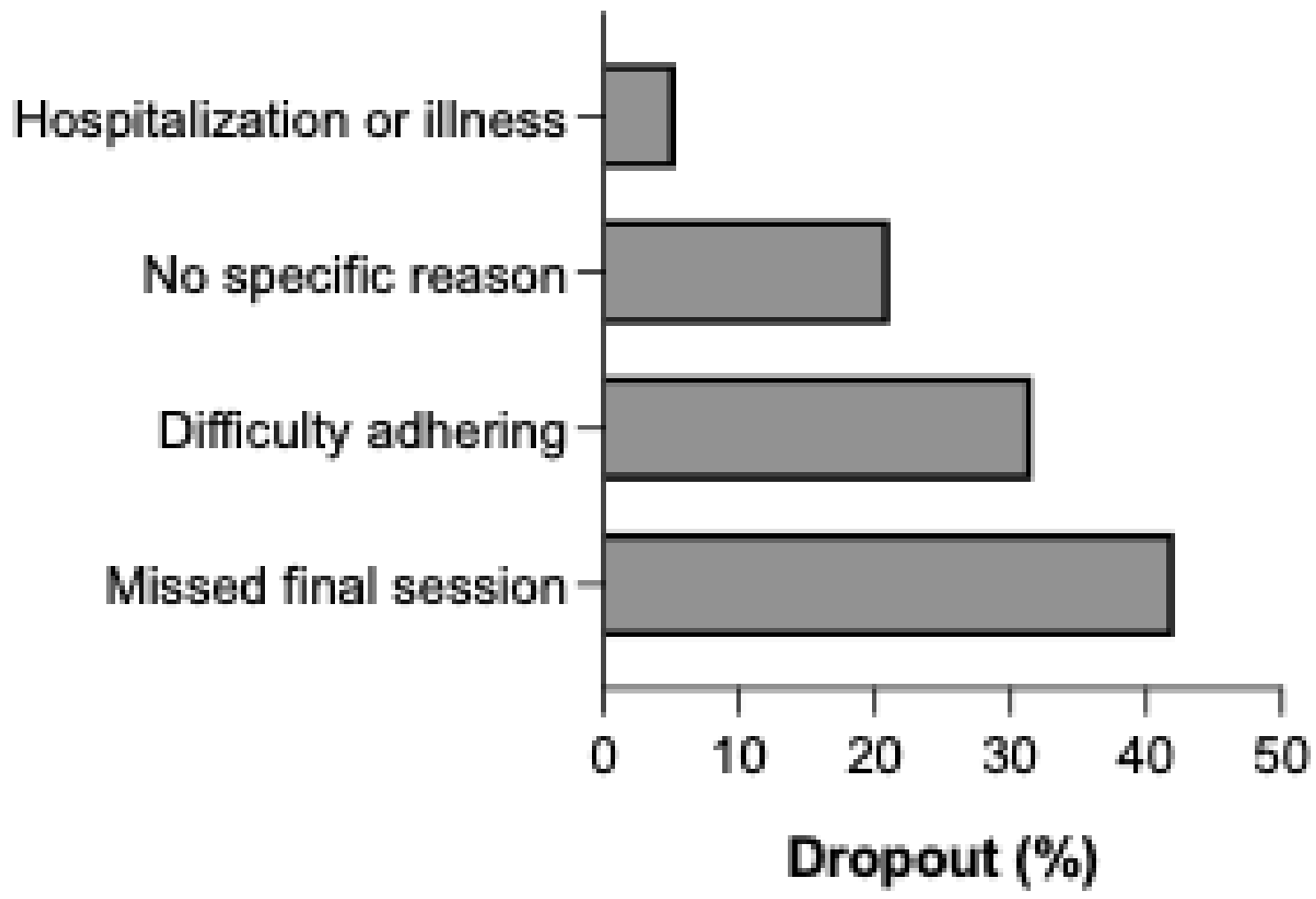

B, Dropout by Generation

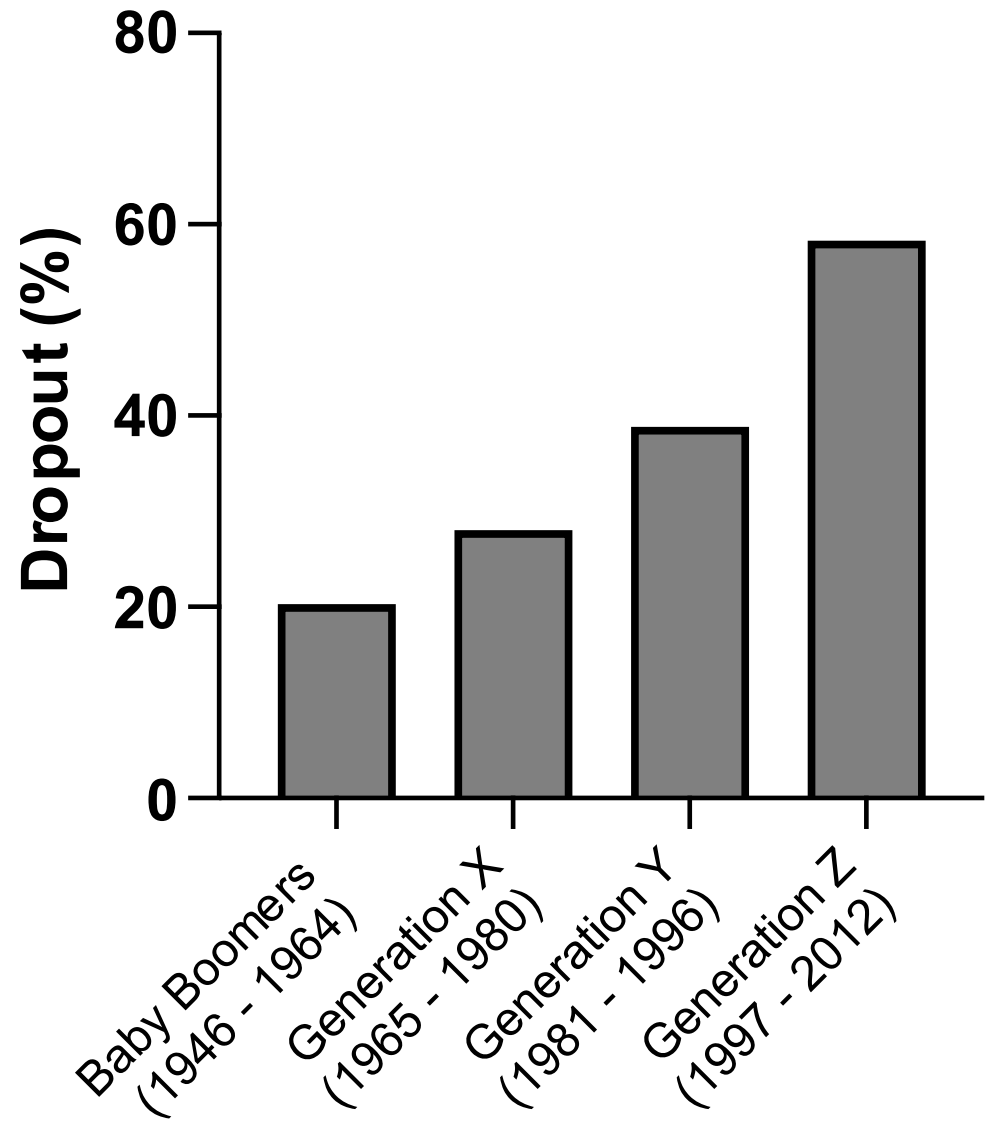

A, Percentage of participants who dropped out of the study, categorized by reason: missed final session, difficulty adhering, no specific reason, or hospitalization/illness.

B, Percentage of participants who dropped out of the study, stratified by generation: Baby Boomers (1946–1964), Generation X (1965–1980), Generation Y (1981–1996), and Generation Z (1997–2012). Dropout rates are shown as percentages of the total number of participants in each category.

© 2026 Souza de Menezes Amorim Coelho G et al. *JAMA Network Open*.

Usual medical care

All participants were already under the care of the endocrinology team at the outpatient clinic of the Hospital das Clínicas, and this care was maintained throughout the study. Patients received pharmacological treatment for obesity and related comorbidities, based on individual clinical needs, as assessed by the resident physician and their supervising endocrinologists. Anti-obesity medications were dispensed by the hospital pharmacy and included sibutramine, topiramate, orlistat, and liraglutide.

The usual medical care protocol at the outpatient clinic included basic guidance on healthy eating and encouragement to engage in physical activity. However, no quantitative nutritional recommendations were provided, and qualitative advice was not based on a standardized protocol. Referrals to the clinic's nutrition team or psychological services occurred only when deemed clinically necessary.

© 2026 Souza de Menezes Amorim Coelho G et al. *JAMA Network Open*.
