# Supplementary material for: A Hybrid Lifestyle Program and Metabolic Health in Adults With Obesity: The PRAMEV Randomized Clinical Trial
Source: JAMA Netw Open. 2026 Aug 11;9(8):e2626884. doi: 10.1001/jamanetworkopen.2026.26884 (PMC13463168; doi:10.1001/jamanetworkopen.2026.26884)
Supplement: Supplementary file 3 [file jamanetwopen-e2626884-s003.pdf]

Data Sharing Statement

Souza de Menezes Amorim Coelho. A Hybrid Lifestyle Program and Metabolic Health in Adults With Obesity. *JAMA Netw Open*. Published August 11, 2026.
doi:10.1001/jamanetworkopen.2026.26884

Data
Additional Information: Name of registry: Brazilian Clinical Trials Registry (ReBEC) Trial registry identifying number: RBR-10wpdd9c; UTN: U1111-1273-8313 URL to registry record: https://ensaiosclinicos.gov.br/rg/RBR-10wpdd9c
Data available: Yes
Data types: Deidentified participant data
How to access data: Data can be requested by contacting the corresponding author (graziele.amc@gmail.com). Requests must include a proposal describing the intended use and will require a signed data use agreement.
When available: With publication

Supporting Documents
Document types: Informed consent form
How to access documents: Data can be requested by contacting the corresponding author (graziele.amc@gmail.com). Requests must include a proposal describing the intended use and will require a signed data use agreement.
When available: With publication

Additional Information
Who can access the data: Researchers whose proposed use of the data has been approved by the study team.
Types of analyses: Systematic reviews, meta-analyses, secondary analyses, and methods development
Mechanisms of data availability: Data will be made available without additional support from the study team and only after a data use agreement has been signed.
